# The Formylation of *N*,*N*‑Dimethylcorroles

**DOI:** 10.1021/acsomega.5c05661

**Published:** 2025-10-01

**Authors:** Sara Nardis, Alessia Fata, Francesco Pizzoli, Greta Imbesi, Greta Petrella, Daniel O. Cicero, Frank R. Fronczek, Kevin M. Smith, Roberto Paolesse

**Affiliations:** † Department of Chemical Sciences and Technologies, 9318 University of Rome Tor Vergata, Via della Ricerca Scientifica, Rome 00133, Italy; ‡ Department of Chemistry, Louisiana State University, Baton Rouge, Louisiana 70803, United States

## Abstract

The chemistry of *N*-alkylcorroles is almost unexplored, although it could represent a promising route for further tuning the properties of this porphyrinoid. Herein, we report our investigations on the β-formylation of *N*(21),*N*(22)-dimethyl-5,10,15-tritolylcorrole, showing how different regioisomers can be obtained by modifying the functionalization reaction sequence. β-Formyl-*N*(21),*N*(22)-dimethyl-5,10,15-tritolylcorrole shows unprecedented reactivity toward acetone in basic conditions, affording a conjugated vinyl ketone derivative in good yields. All these products feature intense absorption bands in the NIR region, which are interesting optical properties with potential applications in different fields.

## Graphical abstract

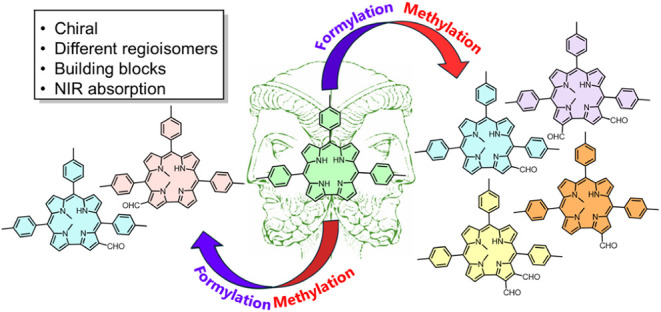

## Introduction

Corroles, a subset of the porphyrinoid macrocycle family, have gained significant attention in recent years due to their unique chemistry, which distinguishes them from their other relatives. Following the introduction of alkylcorroles by Johnson and Kay in 1965,^1^ a key milestone in their chemistry was reached in the early 21st century, when straightforward synthetic methods for accessing *meso*-aryl derivatives were developed.^2–5^ This advance led to an increased focus on corroles and their metal complexes, enabling more extensive studies and revealing promising potential applications for these compounds.^6,7^

Despite much progress, several aspects of corrole chemistry continue to be both challenging and intriguing. For instance, the coordination of divalent ions to the corrole ligand is difficult due to its trianionic nature. Additionally, the reactivity exhibited is often unexpected compared with the parent porphyrins owing to the less symmetric corrole structure, greater tendency to undergo oxidation, and peculiar acid–base characteristics. Among the possible approaches adopted over the years, a promising solution to the divalent ion metalation challenge lies in investigating the coordination behavior of *N*-substituted corroles. N-Alkylation, achieved either through one-pot synthesis^8^ or by postfunctionalization of the macrocycle,^9^ is known to significantly influence the properties of porphyrinoids. After being studied for the functionalization of β-alkylcorroles,^10^ this approach has been used to facilitate the formation of triarylcorrole dianionic ligands, enabling the coordination of ions such as Pd­(II),^11^ Rh­(I),^12^ and Zn­(II).^12^

Despite its considerable potential, *N*-alkylation remains one of the least explored functionalizations in corrole chemistry. It is worth noting that in addition to transforming corroles into dianionic ligands, N-alkylation reduces their acidity, influences their stability and solubility, induces a notable red shift of their Q bands in the near-infrared region (a feature potentially valuable for various applications), and enables the isolation of chiral products—something not feasible with the free base due to the rapid tautomeric exchange inherent to corrole macrocycles.^13,14^ Moreover, the size of the alkyl group and the number of alkyl substituents can be easily tuned using different synthetic approaches, allowing for the preparation of a broad range of *N*-substituted corrole derivatives.^15^

Given the growing interest in the applications of corroles, it is important to explore how these derivatives can be further functionalized by introducing specific groups at their peripheral positions to allow their anchoring on solid substrates, for example, to modify their solubility or optical characteristics.

In the present study, we focused on the β-functionalization of *N*-alkylated corroles to investigate how the alkyl group influences the regioselectivity of the reaction and to assess potential differences in terms of reactivity compared to the unmodified arylcorroles. Except for halogenation,^16–22^ the β-functionalization^23^ of arylcorroles is rarely complete, usually leading to complex mixtures of differently substituted compounds with the potential formation, in contrast to porphyrins, of a variety of regioisomers. While this can present challenges in terms of purification and reaction yields, it also offers exciting opportunities for further exploration, in particular in the case of *N*-substituted corroles for which this effect could be amplified. It is important to note that the presence of substituents in the inner core further reduces the symmetry of the contracted porphyrin analog, thus making further functionalization more challenging due to the formation of a huge number of possible isomers.

Among the various functionalization reactions developed for arylcorroles,^23^ we chose to study formylation because it can easily introduce an easily modified carbon atom at the peripheral positions of the macrocycle, for example, through the Knoevenagel reaction.^24–29^

The Vilsmeier–Haack procedure is the reaction of choice for the formylation of tetrapyrroles in porphyrinoid chemistry.^29–31^ Previous studies on its application to *meso*-arylcorroles have demonstrated that the reaction is quite regioselective, with the primary product being the 3-formylcorrole, and byproducts arising from attack of the Vilsmeier reagent to the macrocycle inner core.^24^ We hypothesized that introducing one or more methyl groups into the inner core could prevent the formation of these byproducts and potentially modify the regioselectivity of the reaction. In *meso*-arylcorroles, the substitution is generally oriented to the directly linked pyrroles (A and D, in Figure 1), while the other two pyrrole subunits (B and C) are much less reactive.^23^ The inclusion of alkyl substituents into the corrole inner core could further refine this regioselectivity by altering the reactivity of the normally more reactive pyrrole subunits.

**Figure 1:**
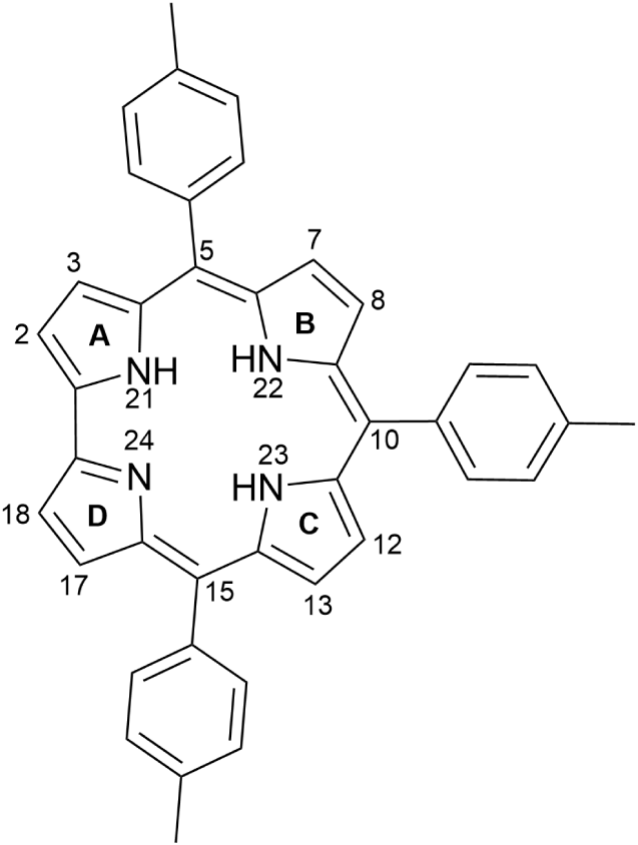
Molecular structure of 5,10,15-tris­(4-methylphenyl)­corrole, **1**.

For this study, we selected 5,10,15-tris­(4-methylphenyl)­corrole (TTC) **1** as the starting substrate, due to its stability, ease of synthesis, and comparable reactivity to other corroles described in the literature,^4^ and we then focused on the preparation and functionalization of *N*(21),*N*(22)-dimethyl derivative instead of the monosubstituted *N*-methylcorroles,^15^ with the aim to limit as much as possible the number of products that could be obtained at this stage, dividing the corrole ring in two sections: the upper part, with the pyrroles A and B, functionalized with the methyl groups, and the bottom part, with the unsubstituted pyrroles C and D. The study involved two different functionalization strategies: (I) *N*(21),*N*(22)-dimethylation followed by the Vilsmeier–Haack reaction, and (II) the Vilsmeier–Haack reaction followed by the N,N-dimethylation. By comparing the products obtained from these two approaches, we aimed to elucidate the distinct effects of the methyl and formyl groups on the reactivity of the corrole macrocycle, providing valuable insights into the role of alkylation in corrole chemistry.

## Results and Discussion

The Vilsmeier–Haack reaction has already been used for the functionalization of corroles to study their reactivity, but also to show how the introduction of different groups onto the corrole scaffold can influence their photophysical behavior.^29,32^

In this case we focused on the *N*21,*N*22-dimethyl-TTC **2** to study the effect of the core functionalization on the β-pyrrolic positions reactivity (Scheme 1): in this corrole both the directly linked (A and D) and the other two pyrroles (B and C) are different due to the presence of the methyl groups.

**Scheme 1:**
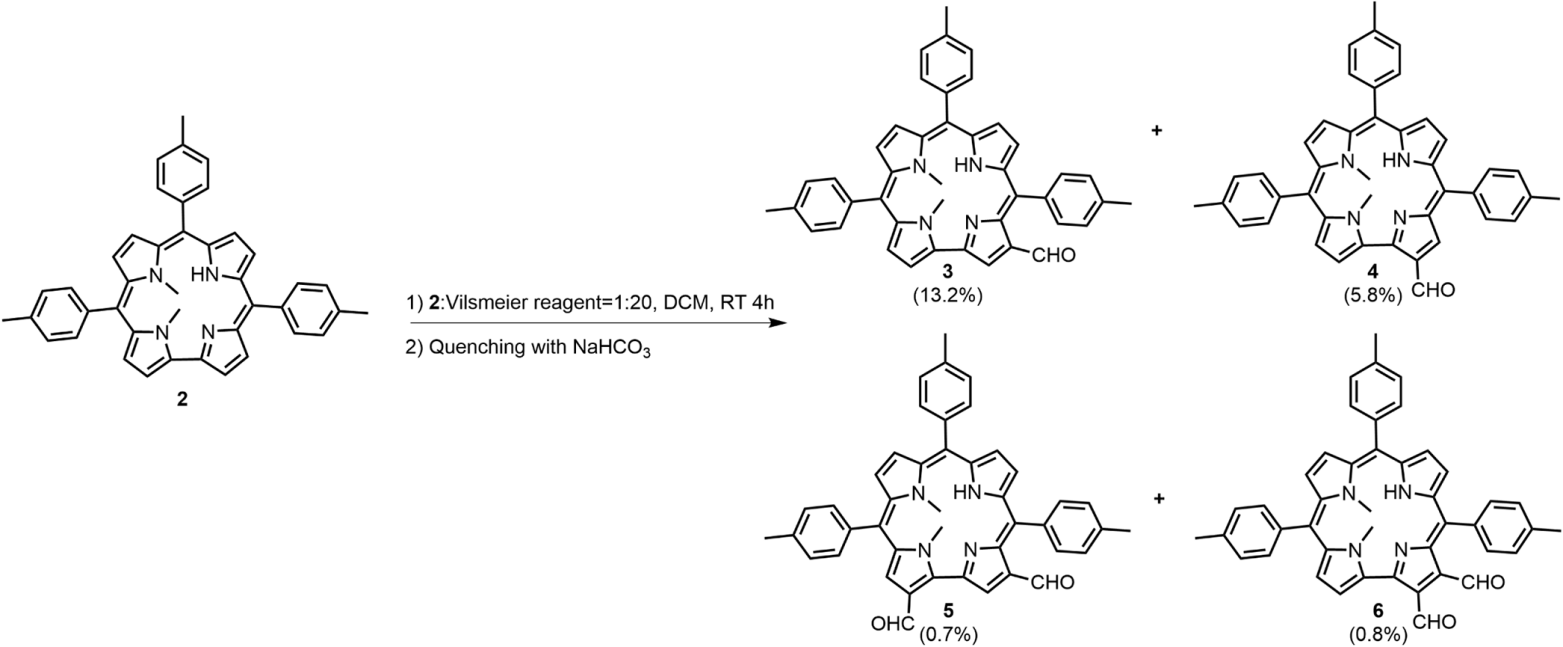
Vilsmeier–Haack Reaction of **2**

The reaction was carried out using the conditions already reported for the formylation of 5,10,15-triphenylcorrole,^24^ but with a reduced excess of the Vilsmeier reagent (corrole 2 to Vilsmeier reagent ratio 1:20), with the aim to avoid as much as possible poly formylation products and determine, by the characterization of the monoformyl derivative, the most reactive position on the skeleton; the formylation reagent ratio adopted was the lowest possible, because a further reduction led to the failure of macrocycle functionalization. The Vilsmeier reagent (POCl_3_/DMF) was added to a solution of the macrocycle in dichloromethane, and the resulting mixture was stirred at room temperature under nitrogen. The progress of the reaction was monitored using UV/vis spectrophotometry and thin layer chromatography (TLC). After 4 h, changes in the UV/vis profile and TLC analysis indicated the formation of different derivatives; the reaction was stopped and quenched overnight by addition of a saturated aqueous solution of NaHCO_3_, necessary to hydrolyze the imine intermediate. From the chromatographic purification, four fractions were isolated and characterized. The major products were promptly characterized by mass spectrometry as the monosubstituted derivatives; a third more polar fraction was purified by an additional chromatographic separation, to afford two additional fractions attributed to the diformylated products, again by mass spectrometry. At this stage, it was crucial to determine the position of the formyl group in the monosubstituted compounds to understand how the inner core methyl groups influenced the reactivity of the macrocycle.

The presence of a formyl substituent was evident in the ^1^H NMR spectra of both products. For one regioisomer, we were able to obtain crystals suitable for X-ray characterization, which allowed its unambiguous characterization as the corrole **3**, with the formyl group introduced at the 17 position (Figure 2).

**Figure 2:**
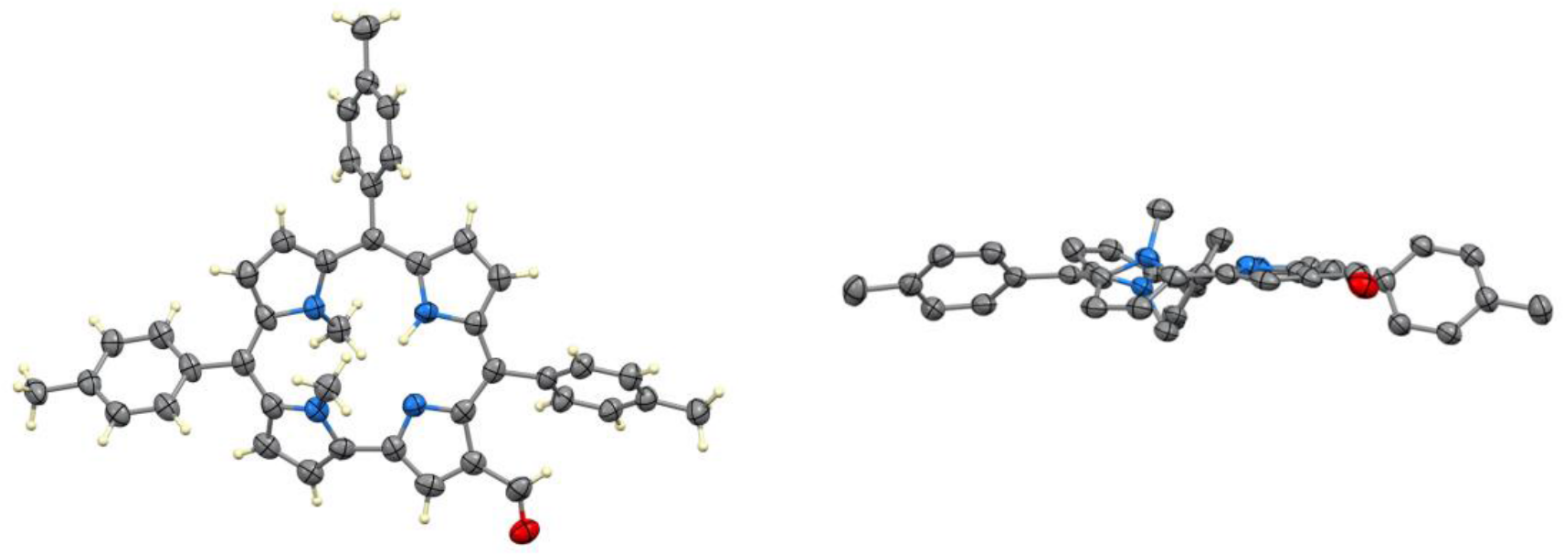
X-ray structure (top-view and side-view) of one of two independent molecules of **3**, with 50% ellipsoids.

The presence of methyl groups on adjacent N atoms causes the corrole core of **3** to be quite nonplanar. The two independent molecules in the crystal have similar shapes. Deviations of the 23 core atoms from a common plane average 0.24 Å over the two molecules, with maximum deviation 0.65 Å. The methyl groups lie on opposite sides of the molecule, with deviations from the central plane 1.55 and 1.79 Å. The four N atoms lie an average of 0.15 Å from a common plane, and the pyrrole rings tilt from this plane by varying degrees. The pyrroles that do not carry methyl groups tilt from the N_4_ plane by a mean dihedral angle of 18.0°, while the alkylated ones are much more strongly inclined, having a mean dihedral angle of 41.9°. The formyl groups tilt slightly out of their pyrrole rings, with mean C–C–C=O torsion angles 18.6°.

A combination of 2D NMR experiments allowed the complete assignment of all the H and C nuclei of compound **3**. Figure 3 shows the aromatic region of the ^1^H NMR spectrum of compound **3** recorded at three different temperatures (*T* = 283, 298, and 313 K). All doublets corresponding to the β-pyrrolic hydrogens are sharp and unaffected by temperature. In contrast, the signals of the *ortho* and *meta* protons of the three *meso*-substituted phenyl rings exhibit markedly different line widths that are dependent upon temperature. At low temperature (*T* = 283 K), distinct exchange regimes are observed for the ortho protons: the phenyl group at position 5 displays a single sharp signal, consistent with a fast exchange regime on the NMR time scale; the phenyl ring at position 10 shows broad and partially coalesced signals, characteristic of intermediate exchange; and the phenyl ring at position 15 exhibits two well-resolved doublets, indicating slow exchange, with restricted rotation rendering the two ortho protons magnetically inequivalent.

**Figure 3:**
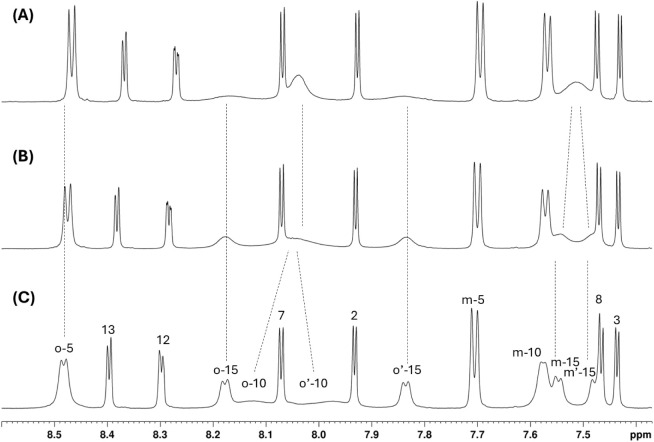
Comparison of ^1^H NMR aromatic regions of compound **3** acquired at 313 K (A), 298 K (B), and 283 K (C), in CDCl_3_.

Upon increasing the temperature, the rate of rotation around the *meso*–aryl bonds increases, driving a progressive transition from slow to intermediate to fast exchange regimes. As a result, the *ortho* signals of the phenyl group at position 15 broaden and approach coalescence, those of the ring at position 10 pass through a coalescence point and collapse into a single signal, and the signals of the phenyl at position 5 become even narrower. This behavior can be rationalized by considering that the observed NMR line width is governed by the ratio between the exchange rate constant (*k*) and the chemical shift difference (Δν) between exchanging sites: in the slow exchange regime (*k* ≪ Δν), separate signals are observed; in the fast exchange regime (*k* ≫ Δν), a single averaged signal appears; and in the intermediate regime (*k* ≈ Δν), broadening and coalescence occur.

The markedly slower rotation of the phenyl ring at position 10 compared to that at position 5 is consistent with previous findings from our group,^33^ which attributed the reduced exchange rate to unfavorable local geometry within the corrole core. In particular, N-methylation of the inner NH introduces macrocyclic distortion that may contribute to a higher rotational barrier at position 10. In contrast, the extremely slow rotation observed for the aryl group at position 15 can be attributed to additional steric hindrance introduced by the formyl substituent at position 17, which restricts conformational freedom in that region of the molecule.

We were unable to obtain crystals of the second regioisomer; however, the 2D NMR experiments enabled us to characterize it as compound **4**, which has the formyl group at position 18. The position of the formyl substituent in compound **4** was determined through a combination of NOESY and HMBC NMR experiments. As shown in Figure 4A, the aldehydic proton shows NOE correlations with two β-pyrrolic protons: a singlet and a doublet. This spatial proximity is only compatible with a geometry in which the formyl group is located on the side of the corrole macrocycle lacking a *meso* carbon bridge, thus narrowing the possible positions to either 2 or 18. To distinguish between these two possibilities, HMBC correlations were analyzed, as illustrated in Figure 4B. The singlet β-pyrrolic proton α to the formyl group shows long-range couplings to a set of quaternary carbons distinct from those coupled to the *N*-methyl groups, which are attached to the pyrroles flanking position 5. This indicates that the formyl group is not on the same pyrrole as the *N*-methyl substituents, excluding position 2 and confirming its location at position 18. The combined NOE and HMBC connectivities are summarized in Figure 4C, where red arrows indicate NOESY correlations and black arrows indicate HMBC couplings, supporting the unambiguous assignment of the formyl group to position 18.

**Figure 4:**
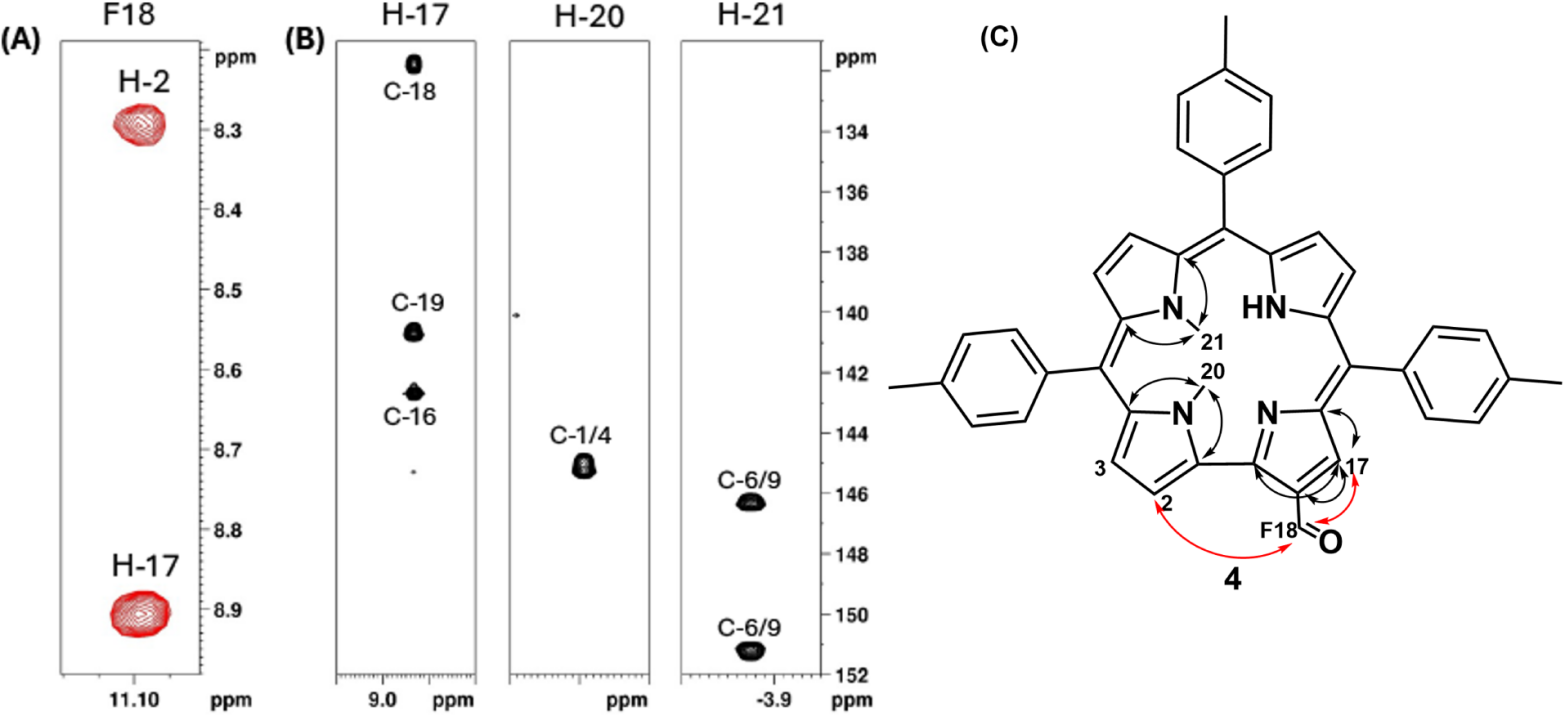
NMR determination of the formyl group position in compound **4**. (A) NOESY correlations indicate proximity of the formyl proton to two β-pyrrolic protons, consistent with substitution at position 2 or 18. (B) HMBC couplings differentiate the quaternary carbon of the formyl-adjacent pyrrole from those of the *N*-methylated pyrroles. (C) Combined NOESY (red) and HMBC (black) correlations support the assignment of the formyl group to position 18.

The downfield region (Figure 5A) highlights a significant difference in the chemical shift of the formyl proton for compound **3** and compound **4**. The formyl hydrogen signal in compound **3** (F17) is strongly shielded compared to that in compound **4** (F18), an effect likely due to the proximity of the formyl group to the anisotropic ring current of the tolyl substituent at position 15, which is spatially more distant in compound **4**. In the aromatic region (Figure 5B), the influence of the formyl group on the dynamics of the tolyl ring at position 15 also differs: in compound **3**, the two *ortho* protons of tolyl-15 at room temperature are clearly resolved as separate signals, while in compound **4** they are close to coalescence, indicating a faster rotational exchange. This suggests that the formyl group at position 17 in compound **3** exerts a greater steric or electronic hindrance on the rotation of the adjacent aryl ring. Additionally, in both compounds, H-12 and H-13 appear as double–doublets due to scalar coupling with the NH, which is bound to pyrrole C and does not undergo tautomerism with pyrrole D. This assignment is supported by COSY (Figure S3A) cross-peaks between the NH signals and H-12/H-13, and by long-range ^1^H–^13^C correlations between the NH and the carbons of pyrrole C in the HMBC (Figure S3B) spectra. In any case, the substitution occurs for both **3** and **4** derivatives at the unmethylated pyrrole D, which appears more reactive than the methylated counterparts.

**Figure 5:**
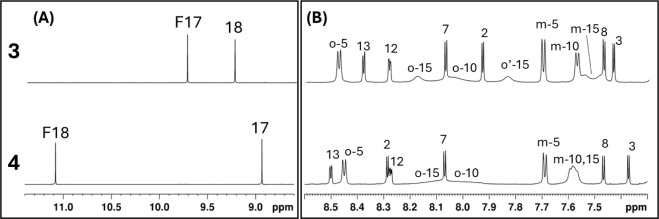
Comparison of the downfield (A) and aromatic (B) regions of the ^1^H NMR spectra of compounds **3** (top) and **4** (bottom) obtained at room temperature. F17 and F18 refer to the formyl hydrogen attached at positions 17 or 18, respectively.

Figure 6 reports the UV–vis spectra of the mono- and bis-formylated derivatives. The profiles are very similar, except for a band in the blue region at 377 nm present for compounds **4** and **5**. The intense absorption in the NIR is an interesting characteristic for all these compounds: compared to the parent corrole, alkylation of the inner core induces the distortion of the macrocycle, leading to a red shift of all absorption bands,^11^ which is further enhanced by the introduction of formyl groups^24^ (Figure S1).

**Figure 6:**
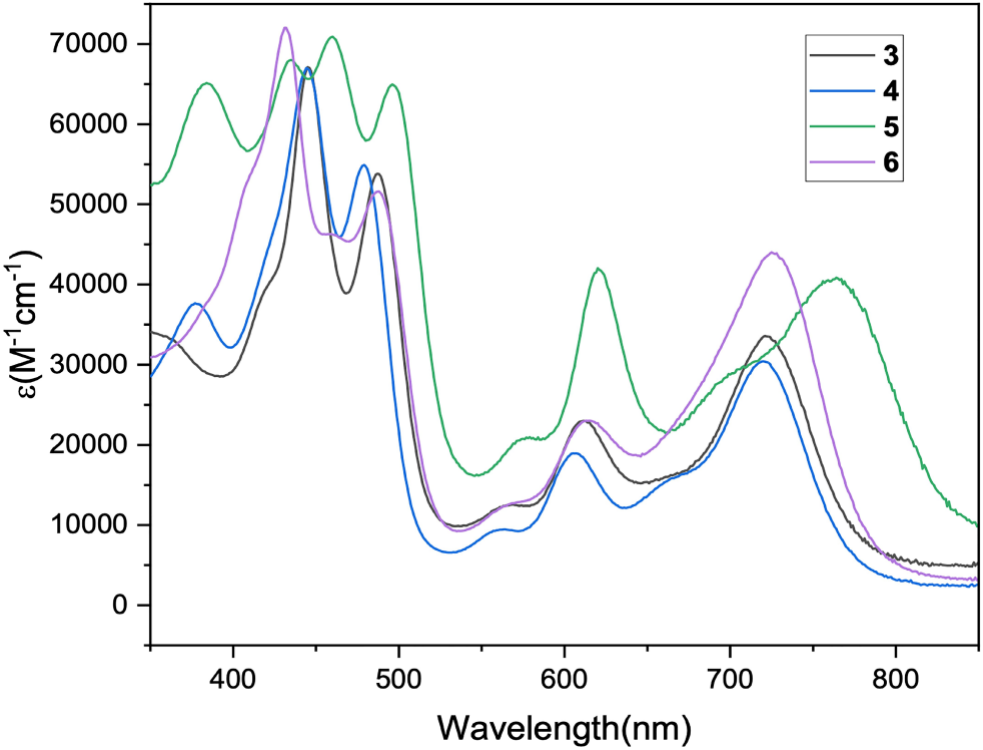
UV–vis spectra in CH_2_Cl_2_ for compounds **3**, **4**, **5**, and **6**.

Notably, the presence of a formyl group in the β-position elucidates the intrinsic chirality of *N*-alkylcorroles; the enantiomers are both present in the crystallographic structure (Figure S11). Confirming this, compound **3** was separated via chiral HPLC into its two enantiomers.

The CD spectra of the two enantiomers in Figure 7 appeared in an almost mirror-image relationship. The spectra show two crossover points corresponding to the Soret band values.

**Figure 7:**
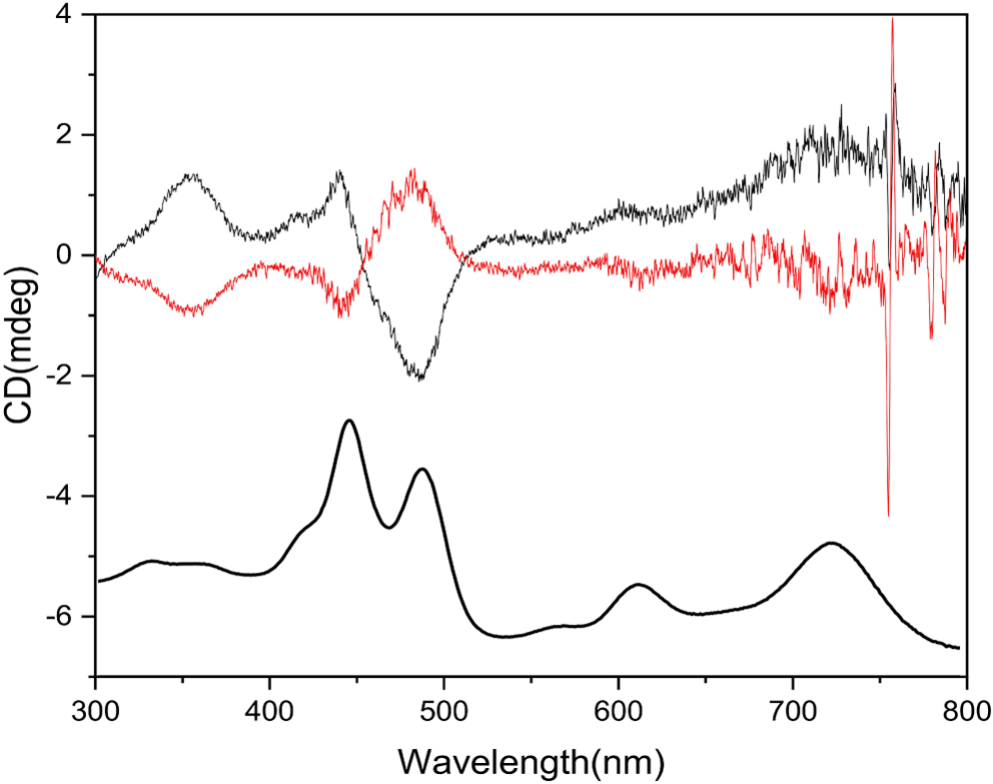
UV–vis and CD spectra in CH_2_Cl_2_ of the first (black line) and second (red line) eluted enantiomers of corrole **3**.

On the other hand, a comparison of the two diformyl compounds **5** and **6** revealed distinct behaviors. The structure of **5**, containing two formyl substituents, was determined through combined analysis of 2D NMR data, including HSQC, HMBC, and NOESY spectra (Figure 8). The first clue arose from the chemical shifts of the two formyl proton signals: one appears at 10.89 ppm, and the other at 9.62 ppm. The latter resembles the formyl resonance observed in **3**, where it is shielded by the nearby tolyl group at position 15. This suggests that the formyl proton at 9.62 ppm is likewise located adjacent to an aryl ring, consistent with substitution at position 17. This assignment is further supported by the NOESY spectrum (Figure 8B), which shows spatial proximity between this shielded formyl proton and the two *ortho* protons of the slowly rotating tolyl ring, confirming that the formyl group is located at position 17. The adjacent β-pyrrolic proton H-18, identified through HMBC and HSQC correlations (Figure 8A), shows a NOESY cross-peak with the other formyl proton (10.89 ppm), indicating that the two are spatially close. This proximity is only possible if the two positions are separated by the bridge that lacks a *meso* carbon, thus locating the second formyl group at position 2. Additionally, the HMBC spectrum reveals that the formyl proton at 10.89 ppm shares a long-range correlation with the same quaternary carbon (C-1) as the *N*-methyl group at position 20, indicating that this formyl group is attached to an *N*-methylated pyrrole subunit, further supporting its assignment to position 2. Finally, the β-pyrrolic proton H-3, assigned via its HMBC and HSQC correlations, shows a NOESY contact with the *ortho* protons of the tolyl group exhibiting the fastest rotation, consistent with its attachment at position 5. Together, these scalar and spatial correlations unambiguously support the assignment of the two formyl groups in **5** to positions 2 and 17.

**Figure 8:**
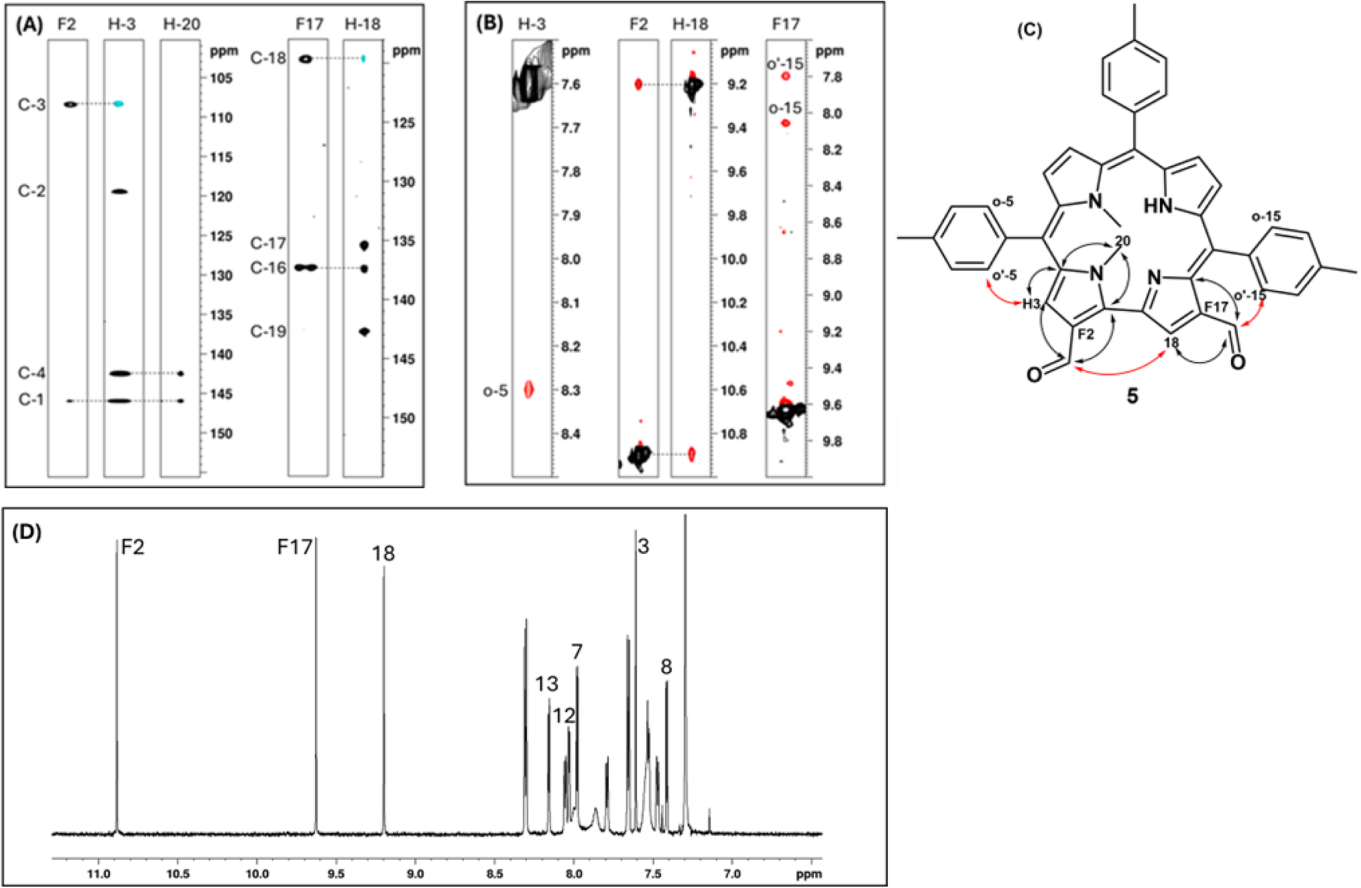
2D NMR data supporting the assignment of formyl groups at positions 2 and 17 in compound **5**. (A) Superposition of HSQC (blue) and HMBC (black); (B) NOESY contacts confirming spatial proximities. (C) Proposed structure for **5**. The arrows indicate the observed connections between the substituent and the macrocycle in the ROESY (red) and HMBC spectra (black). (D) 1D NMR data with β-pyrrolic protons assignment.

The same 2D NMR characterization for **6** could not be performed, because in the experiment time scale, the compound was not stable and the NMR spectra changed over time. The final product did not show the formyl resonances, but it retained a corrole structure, as demonstrated by the UV–vis spectrum (Figure S2). While the small amount of the sample did not allow a detailed characterization of the product, the observed reactivity is interesting, and it will be the object of future study.

Nevertheless, a tentative characterization of **6** bearing two formyl groups at positions 17 and 18 can be proposed based on the ^1^H NMR data shown in Figure 9. The downfield region (Figure 9A) displays two singlets attributable to formyl protons. One appears significantly upfield, similar to the chemical shift observed for formyl groups at position 17 in related compounds, suggesting shielding from a nearby aryl ring. The aromatic region (Figure 9B) shows six distinct doublets corresponding to β-pyrrolic hydrogens, consistent with a substitution pattern in which both formyl groups are located on the same pyrrole. Given the regioselectivity observed in monoformylated analogs and the diformylated compound **5**, where ring D is preferentially functionalized, we infer that both formyl groups in **6** are most likely attached to pyrrole D, at positions 17 and 18. Notably, the signals corresponding to the *ortho* protons of the three *meso*-aryl substituents exhibit a pattern of slow, intermediate, and fast exchange regimes, analogous to observations for compounds **3** and **4**, providing further support for a structurally related framework. Based on these combined observations, a putative assignment of the proton signals is proposed.

**Figure 9:**
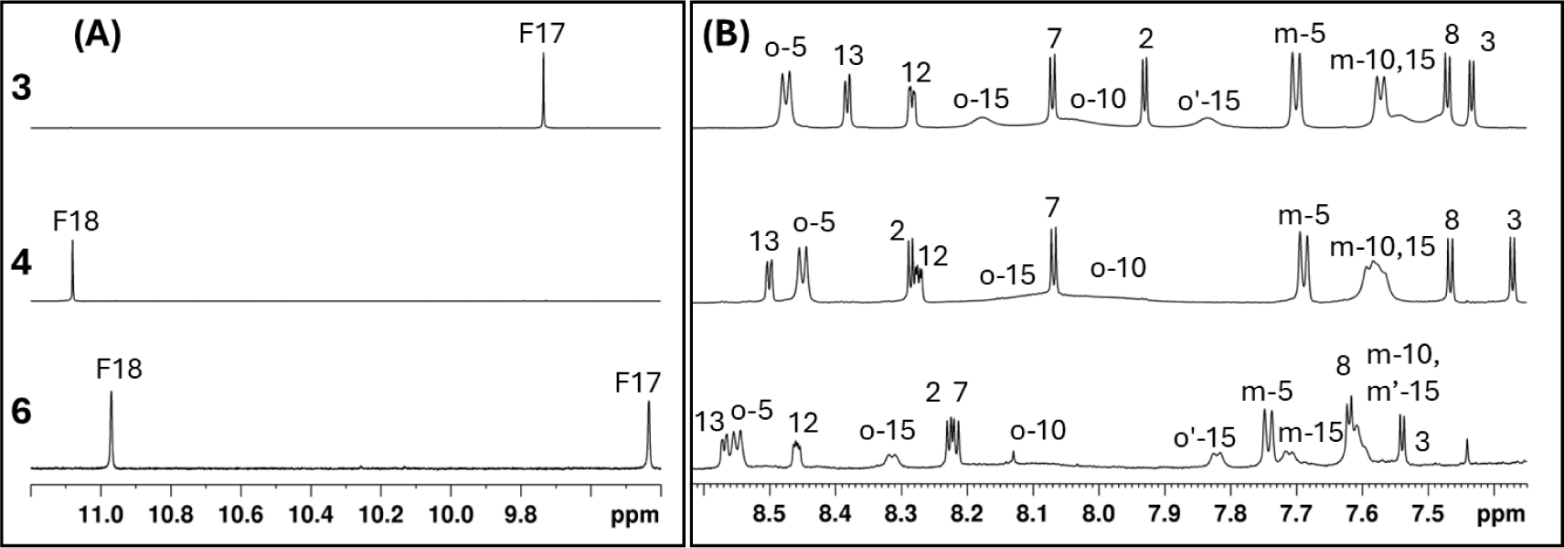
^1^H NMR spectra of compound **6** compared with compounds **3** and **4**. (A) The downfield region shows two singlets consistent with two formyl groups; one is significantly shielded, suggesting proximity to an aryl ring. (B) The aromatic region shows six β-pyrrolic doublets, indicating substitution on the same pyrrole. The figure shows a proposed assignment based on compounds **3** and **4**.

The data collected for the diformylated compounds confirmed that N-methylation drives the functionalization on the unsubstituted pyrroles, affording different substitution products.

*N*-alkyl-substituted pyrroles are generally more reactive than unsubstituted pyrroles toward electrophilic aromatic substitution reactions.^34^ In the case of *N*-substituted corroles, the obtained products, both for mono- and disubstituted compounds, support a higher reactivity of the unsubstituted D ring compared to the substituted A ring, corroborating previous findings reported for both *N*-alkylporphyrins^35^ and *N*-alkylcorroles.^36^ In these systems, X-ray crystallographic analysis revealed that the bond length between the β-carbons of the unsubstituted pyrroles is shorter than that of the *N*-substituted pyrrole. This feature could account for the lower reactivity of the methylated pyrrole in electrophilic aromatic substitution reactions.

The second approach of the investigation consisted of the inversion of the reaction steps to compare the resulting products. The formylation of TTC was carried out (Scheme 2), followed by N-methylation.

**Scheme 2:**
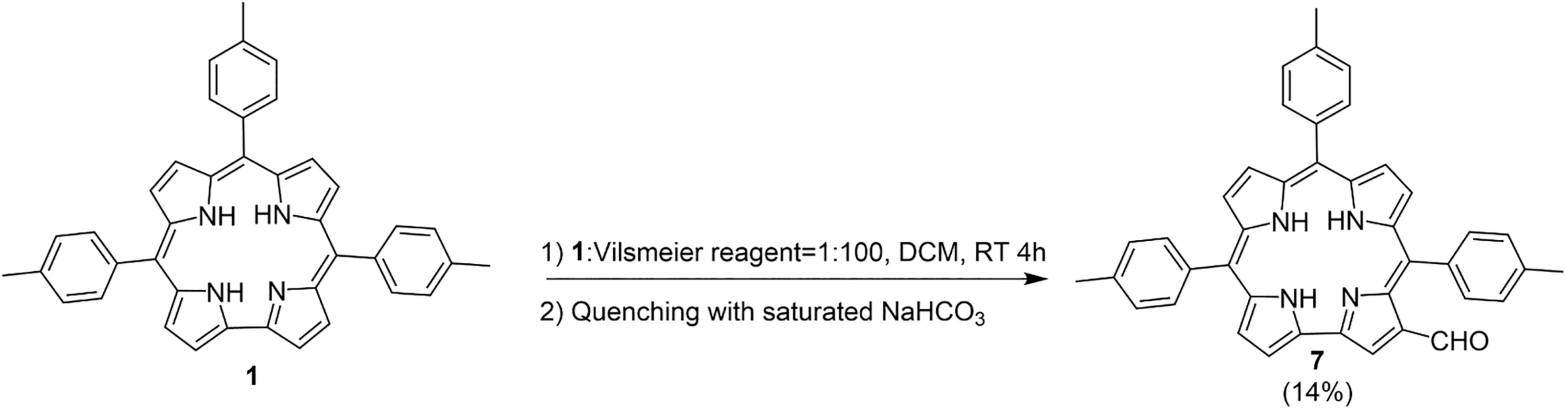
Vilsmeier–Haack Reaction of Compound **1**

Methylation of compound **7** using the Vilsmeier reagent, followed by aqueous quenching, led to the formation of two products (Scheme 3).

**Scheme 3:**
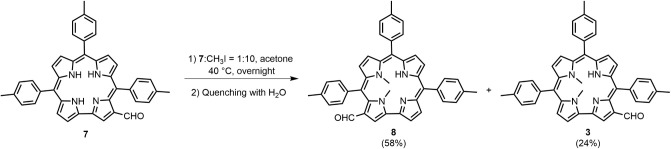
N-Methylation of **7** Quenched with Water

One of them exhibited UV–vis and ^1^H NMR spectra superimposable with those of **3**, confirming its identity. The second product, assigned as compound **8**, was tentatively identified as the isomer bearing the formyl group at position 3, based on spectral comparison and reaction conditions. Although its structure could not be definitively confirmed due to rapid degradation in solution, which prevented acquisition of 2D NMR data, a ^1^H NMR spectrum was successfully recorded. Notably, the yield of compound **8** is approximately twice that of **3**. This difference is likely due to the increased acidity of the formylated tautomer that leads to **8**, which favors its deprotonation and subsequent methylation. The enhanced acidity arises from the presence of the formyl group in the β-position relative to the N(21)–H exerting an inductive electron-withdrawing effect that facilitates proton abstraction.

When the methylation reaction was quenched with 1 M NaOH instead of water, two additional products in addition to compounds **3** and **8** (Scheme 4) were observed.

**Scheme 4:**
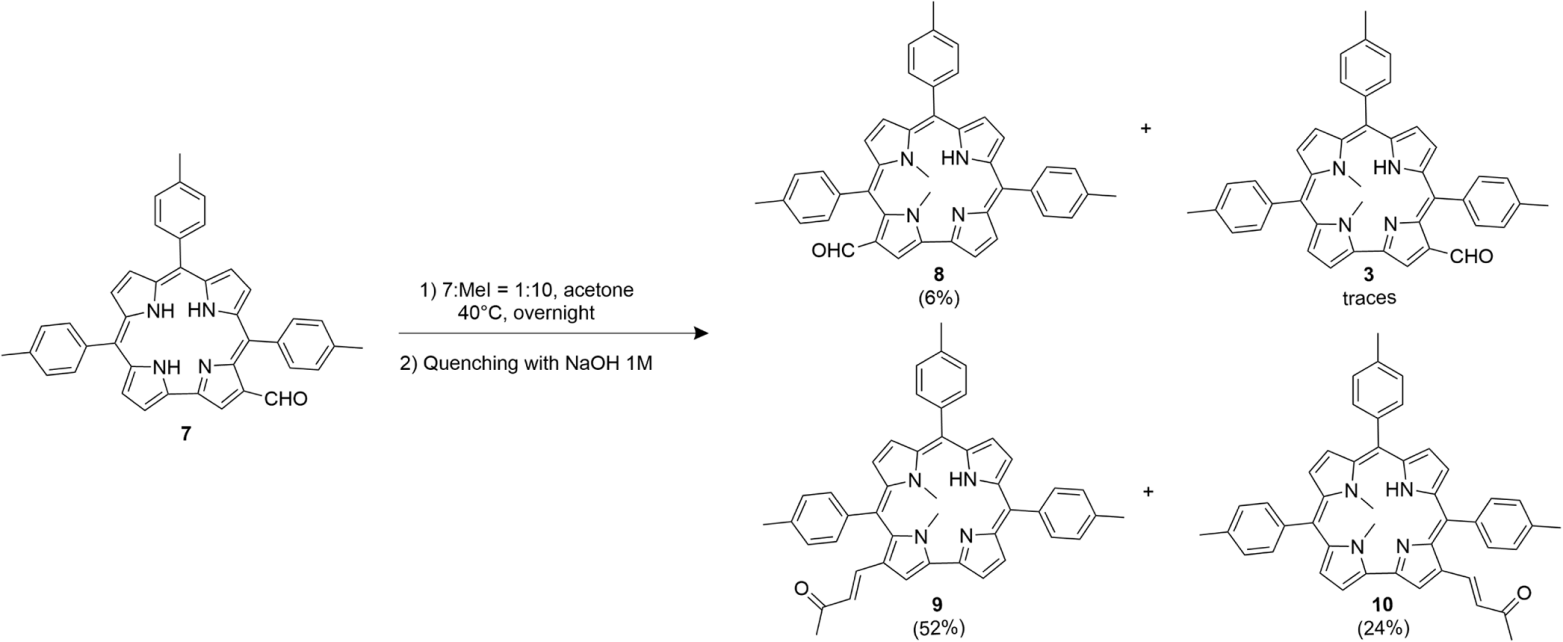
N-Methylation Reaction of Compound **7**

These new compounds lacked the characteristic aldehydic proton signal in their ^1^H NMR spectra. As shown in Figure 10, the spectra revealed two vinylic doublets with a large coupling constant (∼16 Hz) characteristic of a *trans* configuration, a methyl signal around 2 ppm, and, in the corresponding HMBC (not shown), a carbonyl resonance near 200 ppm. These features are consistent with crossed aldol condensation products formed between the formyl group and acetone (the solvent), yielding α,β-unsaturated ketone derivatives (Figure 10A). These products could be of interest in further investigations, so the reaction should be repeated under the same conditions as the N-methylation.

**Figure 10:**
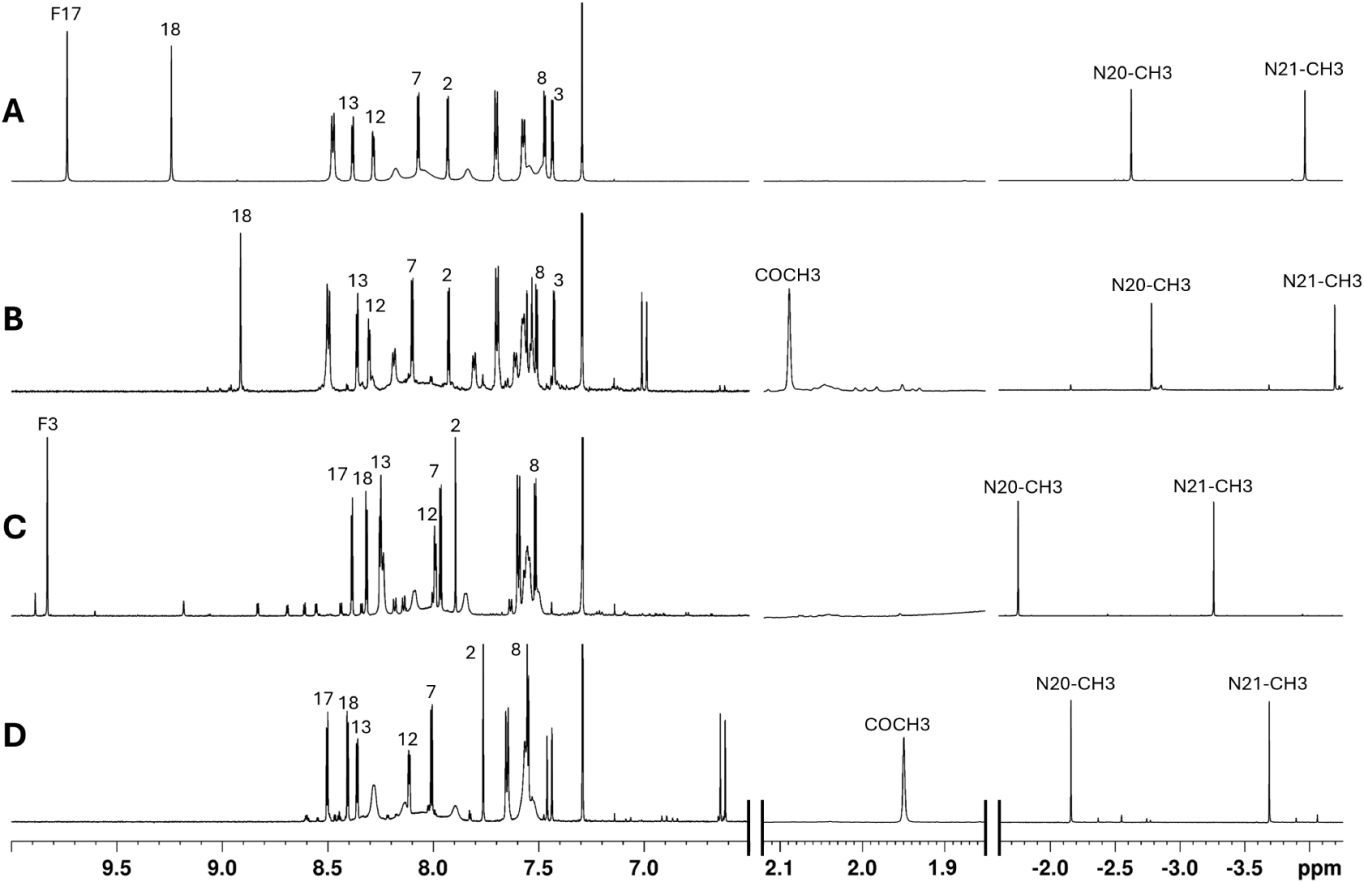
Superposition of ^1^H NMR spectra **3** (A), **10** (B), **8** (C), **9** (D), in CDCl_3_.

Figure 10 shows the superposition of the ^1^H NMR spectra of compounds **3**, **10**, **8**, and **9**. Compounds **3** and **10** display very similar chemical shifts for all shared proton environments, confirming the structural assignment of 10 as proposed in Scheme 4. This assignment was further validated by 2D NMR experiments. Compounds **8** and **9** likewise exhibit closely matching chemical shifts for their common signals, most notably for the β-pyrrolic proton adjacent to the substituent. These similarities suggest that **9** is derived from **8** via crossed aldol condensation of the formyl group with acetone, while retaining the same methylation pattern. UV–vis spectra of the four compounds further support the similarities observed by ^1^H NMR (Figure 11).

**Figure 11:**
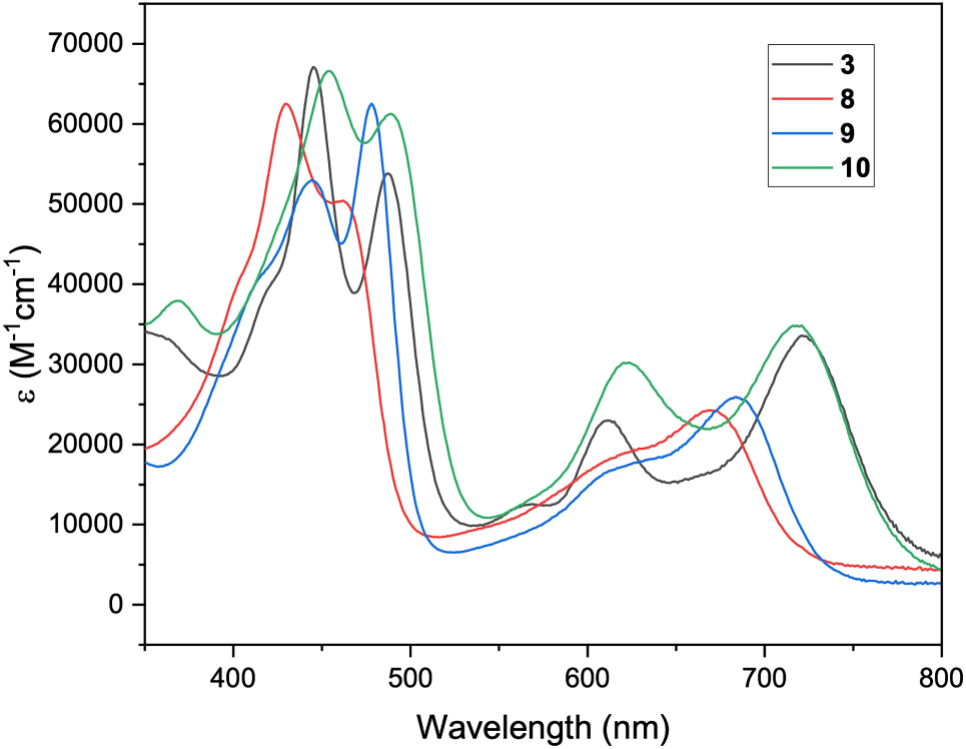
UV–vis spectra, in CH_2_Cl_2_, of compounds **3**, **8**, **9**, and **10**.

The structure of **9** was definitively established through a combination of 2D NMR experiments. As shown in Figure 12A, key cross-peaks in the HMBC spectrum provided unambiguous evidence for the position of the methyl substituents. In particular, the correlations observed for the proton resonating around −2.2 ppm confirmed its connectivity to carbon signals in the 143–145 ppm range, identifying the methylation site on the pyrrole ring. These diagnostic correlations support the assignment of the substituent to position 3, as indicated in Figure 12B, where the observed long-range couplings are mapped onto the molecular structure. This structural assignment is consistent with the proposed reaction pathway. Compound **9** arises via a crossed aldol condensation from compound **8**. Therefore, the confirmed substitution pattern of **9** validates the proposed structure of **8**, in which the methylation occurred on the pyrrole ring bearing the formyl group.

**Figure 12:**
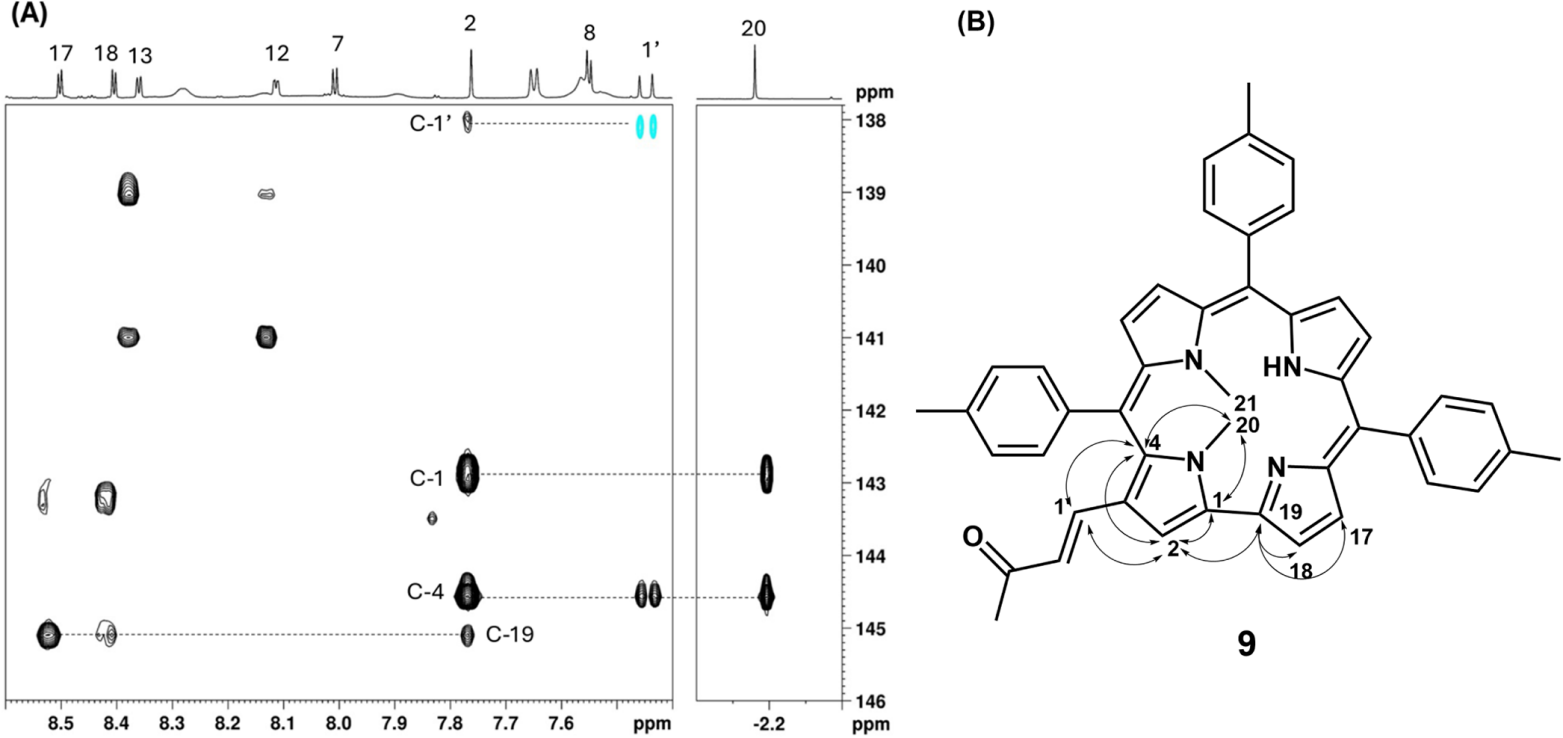
(A) Key HMBC correlations used to assign the methylation pattern in compound **9**. (B) Structure of compound **9** with annotated correlations, confirming substitution at position 3 and supporting the proposed structure derived from compound **8**.

## Conclusions

The nature of the products obtained from the Vilsmeier reaction of *N*-alkylated corroles can be tuned by modification of the functionalization reaction sequence. In this work, we have investigated the functionalization of the *N*(21),*N*(22)-dimethyl-5,10,15-tritolylcorrole **2**, chosen to both reduce the number of potential regioisomers and to divide the corrole ring into two different parts.

When the corrole N-alkylation is first performed, the subsequent formylation is oriented toward the two β*-*positions of the pyrrole D, close to the direct pyrrole–pyrrole link, with a higher yield for the 17-position. The chiral nature of this corrole was evidenced by the resolution of the enantiomeric pair using chiral chromatography. It is interesting to note that **2** is more reactive than the unsubstituted corrole **1**, because it reacted completely with a smaller amount of the Vilsmeier reagent, affording also traces of the diformylated species **5** and **6**. To the best of our knowledge, this last derivative β,β-diformylated in the same pyrrole is unprecedented in the corrole series, although it showed reduced stability in solution.

When the formylation was the first reaction performed, the subsequent N-alkylation was preferentially oriented toward the derivative **8**, the corrole side bearing the formyl substituent, probably as a consequence of the higher acidity of the functionalized pyrrole. It is important to note that these corroles (**3** and **8**) were reactive under basic conditions with the solvent acetone, affording almost quantitatively the corresponding α, β-unsaturated derivatives **9** and **10**.

All these corroles feature interesting optical properties with increased absorption bands in the NIR region, as expected for such formylated derivatives, and for this reason, they are promising as starting materials for future functionalized compounds of interest for different fields of application, such as PDT (Photodynamic Therapy) and NLO (Nonlinear Optics).^29^

## Experimental Section

The syntheses of TTC, **1**, and *N*(21),*N*(22)-dimethyl-TTC, **2**, were carried out according to literature methods.^4,11^

### General Procedure for the Vilsmeier Reaction of **2**

The Vilsmeier reagent was prepared by cooling DMF (0.6 mL, 7.36 mmol) to 0 °C and adding POCl_3_ (0.6 mL, 6.03 mmol) under nitrogen; the reagent was then added dropwise to a solution of **2** (180 mg, 0.30 mmol) in CH_2_Cl_2_ (10 mL). The resulting mixture was allowed to reach room temperature while being stirred under nitrogen. The progress of the reaction was monitored by UV/vis spectroscopy and TLC. After 4 h, a saturated solution of NaHCO_3_ (10 mL) was added and the mixture was stirred overnight; the organic phase was separated, washed with water, dried over anhydrous Na_2_SO_4_, and the solvent was evaporated. The crude mixture was dissolved in CH_2_Cl_2_, and its purification by column chromatography (SiO_2_, CH_2_Cl_2_:hexane 3:1, v/v) afforded **4** as a green band and **3** as a second band. Pure **3** (25 mg, 13.2% yield) and **4** (11 mg, 5.8% yield) were obtained as green crystals after crystallization from CH_2_Cl_2_/MeOH. A third band was eluted using CH_2_Cl_2_/MeOH (9:1, v/v), and then again purified by chromatography on SiO_2_ (hexane: ethyl-acetate 8:2, *v*/*v*), to afford pure **5** (1.3 mg, 0.7% yield) and **6** (1.5 mg, 0.8% yield) as green crystals, after crystallization from CH_2_Cl_2_/MeOH (1:2).

17-Formyl-*N*(21),*N*(22)-dimethyl-TTC, **3**: *R*_f_ = 0.37 (silica, CH_2_Cl_2_/hexane 3:1), UV–Vis (CH_2_Cl_2_)/λ_max_, nm (ε); 444 (66 600), 486 (53 200), 609 (19 700), 719 (31 560). ^1^H NMR (700 MHz, CDCl_3_) δ (ppm) = 9.74 (s, 1H), 9.24 (s, 1H), 8.47 (d, *J* = 8.2 Hz, 2H), 8.37 (d, *J* = 4.6 Hz, 1H), 8.28 (dd, *J* = 4.6 Hz, 2 Hz, 1H), 8.17 (s, 1H), 8.06 (d, *J* = 4.7 Hz, 1H), 7.92 (d, *J* = 4.2 Hz, 1H), 7.83 (s, 1H), 7.69 (d, *J* = 7.7 Hz, 2H), 7.57 (d, *J* = 7.8 Hz, 2H), 7.46 (d, *J* = 4.6 Hz, 1H), 7.43 (d, *J* = 4.1 Hz, 1H), 2.68 (d, *J* = 5.4 Hz, 6H), 2.66 (s, 3H), −2.63 (s, 3H), −3.97 (s, 3H). ^13^C NMR: δ 20.9–21.2 (3C, 21.1 (s)), 25.9 (1C, s), 26.7 (1C, s), 110.0 (1C, s), 110.4 (1C, s), 110.6 (1C, s), 116.1 (1C, s), 118.1 (1C, s), 118.7 (1C, s), 119.3 (1C, s), 119.5 (1C, s), 121.7 (1C, s), 125.9 (1C, s), 127.5 (1C, s), 127.7 (1C, s), 127.8 (2C, s), 127.9 (2C, s), 129.4 (2C, s), 132.1 (1C, s), 134.0 (1C, s), 134.8 (2C, s), 135.4 (2C, s), 136.2 (1C, s), 136.8 (1C, s), 137.5 (1C, s), 137.6 (1C, s), 138.3 (1C, s), 139.3 (1C, s), 141.4 (1C, s), 142.0 (1C, s), 143.2 (1C, s), 144.1 (1C, s), 145.6 (1C, s), 146.6 (1C, s), 151.3 (1C, s), 189.0 (1C, s). HRMS (ESI/TOF): *m*/*z* [C_43_H_37_N_4_O]^+^ (M + H) calculated 625.2967, found 625.2912.

18-Formyl-*N*(21),*N*(22)-dimethyl-TTC, **4**: *R*_f_ = 0.82 (silica, CH_2_Cl_2_/hexane 3:1), UV–Vis (CH_2_Cl_2_)/λ_max_, nm (ε) 377 (35 100), 444 (67 000), 478 (55 700), 605 (17 700), 720 (30 300). ^1^H NMR (700 MHz, CDCl_3_) δ (ppm) = 11.08 (s, 1H), 8.93 (s, 1H), 8.49 (dd, *J* = 4.7 Hz, 1H), 8.45 (d, *J* = 7.6 Hz, 2H), 8.28 (d, *J* = 4.2 Hz, 1H), 8.27 (dd, *J* = 4.6 Hz, 1H), 8.18–7.94 (br, 4H), 8.07 (d, *J* = 4.3 Hz, 1H), 7.69 (d, *J* = 7.9 Hz, 7H), 7.59 (d, *J* = 7.9 Hz, 2H),), 7.57 (d, *J* = 7.9 Hz, 2H), 7.47 (d, *J* = 4.6 Hz, 1H), 7.37 (d, *J* = 4.3 Hz, 1H), 3.42 (s,1H), 2.67 (s, 9H), −2.51 (s, 3H), −3.87 (s, 3H). ^13^C NMR: δ 20.9–21.2 (3C, 21.1 (s)), 28.5 (1C, s), 32.3 (1C, s), 108.8 (1C, s), 110.1 (1C, s), 111.6 (1C, s), 115.7 (1C, s), 120.3 (1C, s), 122.3 (1C, s), 124.9 (1C, s), 126.6 (1C, s), 127.9 (2C, s), 128.1 (2C, s), 129.3 (2C, s), 131.2 (1C, s), 131.9 (1C, s), 133.4 (2C, s), 136.1 (2C, s), 134.7 (2C, s), 136.4 (1C, s), 137.0 (1C, s), 138.0 (1C, s), 138.2 (1C, s), 140.3 (1C, s), 140.6 (1C, s), 142.6 (1C, s), 144.9 (1C, s), 145.2 (1C, s), 146.2 (1C, s), 151.1 (1C, s), 186.9 (1C, s). HRMS (ESI/TOF): *m*/*z* [C_43_H_37_N_4_O]^+^ (M + H) calculated 625.2967, found 625.3000.

2,17-Diformyl-*N*(21),*N*(22)-dimethyl-TTC, **5**: *R*_f_ = 0.33 (silica, hexane: ethyl acetate 8:2), UV–Vis (CH_2_Cl_2_)/λ_max_, nm (ε) 384 (35 000), 432 (67 300), 459 (53 200), 495 (52 700), 619 (30 300), 758 (34 600). ^1^H NMR (700 MHz, CDCl_3_) δ (ppm) = 10.87 (s, 1H), 9.64 (s, 1H), 9.20 (s, 1H), 8.30 (d, *J* = 7.9 Hz, 2H), 8.15 (d, *J* = 4.6 Hz, 1H), 8.05 (d, *J* = 7.4 Hz, 1H), 8.02 (dd, *J* = 4.6 Hz, 1.4 Hz, 1H), 7.98 (d, *J* = 4.8 Hz, 1H), 7.94–7.81 (br, 2H), 7.79 (d, *J* = 7.7 Hz, 1H), 7.65 (d, *J* = 7.8 Hz, 2H), 7.60 (s, 1H), 7.53 (m, 3H), 7.47 (d, 1H), 7.41 (d, *J* = 4.8 Hz, 1H), 2.66 (t, 9H), −1.69 (s, 3H), −2.84 (s, 3H). ^13^C NMR: δ 20.9–21.2 (3C, 21.1 (s)), 30.1 (1C, s), 30.3 (1C, s), 108.3 (1C, s), 117.6 (1C, s), 119.3 (1C, s), 119.6 (1C, s), 123.3 (1C, s), 124.0 (1C, s), 124.6 (1C, s), 126.1 (1C, s), 127.8 (1C, s), 128.0 (2C, s), 128.1 (2C, s), 129.3 (1C, s), 129.5 (2C, s), 132.0 (1C, s), 132.9 (1C, s), 134.4 (2C, s), 134.4 (1C, s), 135.3 (1C, s), 135.4 (1C, s), 135.8 (1C, s), 136.1 (1C, s), 137.3 (1C, s), 138.1 (1C, s), 138.9 (1C, s), 142.2 (1C, s), 142.5 (1C, s), 145.4 (1C, s), 145.9 (1C, s), 147.4 (1C, s), 153.4 (1C, s), 191.8 (1C, s). MS (ESI/TOF): *m*/*z* [C_44_H_37_ N_4_O_2_]^+^ (M + H), calculated 653.29, found 653.23. Found: C, 81.01; H, 5.54; N, 8.53%. C_44_H_36_N_4_O_2_ requires C, 80.96; H, 5.56; N, 8.58%.

17,18-Diformyl-*N*(21),*N*(22)-dimethyl-TTC, **6**: *R*_f_ = 0.38 (silica, hexane:ethyl acetate 8:2), UV–Vis (CH_2_Cl_2_)/λ_max_, nm (ε) 430 (69 500), 485 (52 900), 611 (19 800), 723 (35 600).^1^H NMR (700 MHz, CDCl_3_) δ (ppm) = 11.39 (s, 1H), 9.82 (s, 1H), 8.35 (d, *J* = 7.5 Hz, 1H), 8.32 (d, *J* = 4.2 Hz, 1H), 8.13 (d, *J* = 4.5 Hz, 1H), 8.07 (dd, *J* = 4.8, 2.0 Hz, 1H), 8.03 (d, 1H), 7.93 (d, 1H), 7.91 (d, *J* = 4.7 Hz, 1H), 7.78 (d, 1H), 7.67 (d, *J* = 7.7 Hz, 2H), 7.55–7.50 (br, 2H), 7.54 (d, *J* = 7.8 Hz, 2H), 7.47 (s, 1H), 7.39 (d, *J* = 4.2 Hz, 1H), 7.30 (d, *J* = 4.7 Hz, 1H), 2.67 (s, 3H), 2.65 (d, *J* = 4.6 Hz, 6H), −1.81 (s, 3H), −2.84 (s, 3H). MS (ESI/TOF): *m*/*z* [C_44_H_36_N_4_O_2_]^+^ (M + H) calculated 653.29, found 653.25. Found: C, 80.99; H, 5.53; N, 8.61%. C_44_H_36_N_4_O_2_ requires C, 80.96; H, 5.56; N, 8.58%.

### General Procedure for the Vilsmeier Reaction of **1** ^24^

The Vilsmeier reagent was prepared by cooling DMF (3.5 mL, 45.6 mmol) to 0 °C and adding POCl_3_ (3.5 mL, 37.4 mmol) under nitrogen; the reagent was then added dropwise to a solution of **1** (200 mg, 0.35 mmol) in CH_2_Cl_2_ (10 mL). The resulting mixture was allowed to reach room temperature while stirred under nitrogen. The progress of the reaction was monitored by UV/vis spectroscopy; after one night, there was no evidence of the absorption of the starting material. A saturated solution of NaHCO_3_ (10 mL) was added and the mixture was stirred overnight; the organic phase was separated, washed with water, dried over anhydrous Na_2_SO_4_, and the solvent was evaporated. The crude mixture was dissolved in CH_2_Cl_2_, and column chromatography (SiO_2_, CH_2_Cl_2_) afforded **7** as a green band with traces of a second band. Pure **7** was obtained as green crystals after crystallization from CH_2_Cl_2_/MeOH (30 mg, 14% yield).

### General Procedure for the N-Methylation Reaction of **7**, Quenched in Water

In a 50 mL flask, 20 mg of **7** and 360 mg of K_2_CO_3_ were dissolved in freshly distilled and degassed acetone under nitrogen. The solution was stirred at room temperature for 15 min, 0.35 mmol of CH_3_I were then added at once, and the mixture was refluxed overnight. The progress of the reaction was monitored by UV/vis spectroscopy; after 12 h, there was no evidence of the absorption of the starting material. Then, the mixture was cooled and washed with H_2_O, and the organic phase purified on chromatographic column (silica, CH_2_Cl_2_). Two fractions were collected: **8** (12 mg, 58% yield), and **3** (5 mg, 24%), all as dark green powders.

3-Formyl-*N*(21),*N*(22)-dimethyl-TTC, **8**: *R*_f_ = 0.62 (silica, CH_2_Cl_2_), UV–Vis (CH_2_Cl_2_)/λ_max_, nm (ε) 429 (62 400), 461 (49 400), 669 (21 700). ^1^H NMR (700 MHz, CDCl_3_) δ (ppm) = 9.83 (s, 1H), 8.38 (d, *J* = 4.0 Hz, 1H), 8.32 (d, *J* = 4.0 Hz, 1H), 8.25 (m, 3H), 8.09 (b, 1H), 7.99 (dd,*J* = 4.4 Hz 1H), 8.05–7.92 (b, 1H), 7.96 (d, *J* = 4.9 Hz, 1H), 7.89 (s, 1H), 7.84 (b, 1H), 7.59 (d, *J* = 7.9 Hz, 2H), 7.58–7.53 (m, 3H), 7.52 (d, *J* = 4.9 Hz, 1H), 7.52–7.47 (b, 1H), 2.65 (s, 6H), 2.62 (s, 3H), −1.75 (s, 3H), −3.26 (s, 3H). MS (ESI/TOF): *m*/*z* [C_43_H_37_N_4_O]^+^ (M + H) calculated 625.29, found 625.17. Found: C, 82.68; H, 5.79; N, 8.99%. C_43_H_36_N_4_O requires C, 82.66; H, 5.81; N, 8.97%.

### General Procedure for the N-Methylation Reaction of **7**, Quenched in NaOH

In a 50 mL flask, 20 mg of **7** and 360 mg of K_2_CO_3_ were dissolved in freshly distilled and degassed acetone under nitrogen. The solution was stirred at room temperature for 15 min, 0.35 mmol of CH_3_I were added, and the mixture was refluxed overnight. The progress of the reaction was monitored by UV/vis spectroscopy; after 12 h, there was no evidence of the absorption of the starting material. The mixture was then cooled, treated with 1 M NaOH solution (5 mL), and washed with H_2_O. The organic phase was dried over anhydrous Na_2_SO_4_, filtered, the solvent was evaporated, and the residue was purified by column chromatography (SiO_2_, CH_2_Cl_2_). Four fractions were collected: **8** (1.3 mg, 6%), **3** obtained only in trace amounts, **9** (11.6 mg, 52%), and **10** (5.3 mg, 24%), all as dark green powders.

*E*-3-(3′-oxobut-1′-en-1′-yl)-*N*(21),*N*(22)-dimethyl-TTC, **9**: *R*_f_= 0.38 (Silica, CH_2_Cl_2_), UV–Vis (CH_2_Cl_2_)/λ_max_, nm (ε) 444 (52 500), 478 (62 500), 684 (24 400).^1^H NMR (700 MHz, CDCl_3_) δ (ppm) 8.49 (d, *J* = 4.0 Hz, 1H), 8.40 (d, *J* = 4.0 Hz, 1H), 8.35 (d, *J* = 4.4 Hz, 1H), 8.27 (b, 2H), 8.17–8.09 (b, 1H), 8.11 (dd, *J* = 4.4, 1.5 Hz, 1H), 8.00 (d, *J* = 4.9 Hz, 1H), 7.89 (b, 1H), 7.76 (s, 1H), 7.64 (d, *J* = 7.8 Hz, 2H), 7.59–7.49 (m, 4H), 7.54 (d, *J* = 4.9 Hz, 1H), 7.45 (d, *J* = 16.1 Hz, 1H), 6.62 (d, *J* = 16.1 Hz, 1H), 2.66 (s, 3H), 2.65 (s, 3H), 2.64 (s, 3H), 1.94 (s, 3H), −2.16 (s, 3H), −3.69 (s, 3H). ^13^C NMR: δ 18.2 (1C, s), 20.9–21.2 (3C, 21.1 (s)), 29.6 (1C, s), 29.7 (1C, s), 103.5 (1C, s), 111.0 (1C, s), 113.8 (1C, s), 115.1 (1C, s), 118.0 (1C, s), 122.9 (1C, s), 123.8 (1C, s), 123.9 (1C, s), 124.6 (1C, s), 125.8 (1C, s), 127.8 (1C, s), 127.9 (1C, s), 128.1 (2C, s), 129.3 (2C, s), 129.6 (1C, s), 134.9 (2C, s), 135.5 (2C, s), 138.1 (1C, s), 139.0 (1C, s), 141.0 (1C, s), 142.9 (1C, s), 143.2 (1C, s), 144.5 (1C, s), 145.1 (1C, s), 148.8 (1C, s), 149.3 (1C, s), 194.4 (1C, s). MS (ESI/TOF): *m*/*z* [C_46_H_41_N_4_O]^+^ (M + H) calculated 665.33, found 665.20. Found: C, 83.05; H, 6.09; N, 8.46%. C_46_H_40_N_4_O requires C, 83.10; H, 6.06; N, 8.43%.

*E*-17-(3′-oxobut-1′-en-1′-yl)-*N*(21),*N*(22)-dimethyl-TTC, **10**: *R*_f_= 0.35 (silica, CH_2_Cl_2_), UV–Vis (CH_2_Cl_2_)/λ_max_, nm (ε) 454 (66 600), 489 (60 700), 622 (28 400), 718 (31 100).^1^H NMR (700 MHz, CDCl_3_) δ (ppm) 8.91 (s, 1H), 8.50 (d, *J* = 7.4 Hz, 2H), 8.36 (d, *J* = 4.5 Hz, 1H), 8.30 (d, *J* = 4.5 Hz, 1H), 8.19 (d, *J* = 7.2 Hz, 1H), 8.15–7.96 (b, 1H) 8.10 (d, *J* = 4.7 Hz, 1H), 7.93 (d, *J* = 4.1 Hz, 1H), 7.80 (d, *J* = 7.3 Hz, 1H), 7.70 (d, *J* = 7.7 Hz, 2H), 7.61 (d, *J* = 7.3 Hz, 1H), 7.57 (d, *J* = 6.7 Hz, 2H), 7.56–7.52 (b, 1H), 7.54 (d, *J* = 16.2 Hz, 1H), 7.51 (d, *J* = 4.7 Hz, 1H), 7.43 (d, *J* = 4.1 Hz, 1H), 7.00 (d, *J* = 16.2 Hz, 1H), 2.68 (b, 9H), 2.09 (s, 3H), −2.78 (s, 3H), −4.20 (s, 3H). ^13^C NMR: δ 20.9–21.2 (3C, 21.1 (s)), 24.6 (1C, s), 28.8 (1C, s), 29.6 (1C, s), 109.6 (1C, s), 110.6 (1C, s), 114.3 (1C, s), 115.5 (1C, s), 119.6 (1C, s), 119.8 (1C, s), 121.2 (1C, s), 125.4 (1C, s), 126.4 (1C, s), 127.6 (2C, s), 127.7 (2C, s), 129.3 (2C, s), 132.2 (1C, s), 133.9 (1C, s), 134.6 (1C, s), 135.6 (1C, s), 136.5 (1C, s), 137.4 (1C, s), 137.8 (1C, s), 140.0 (1C, s), 141.4 (1C, s), 141.5 (1C, s), 144.4 (1C, s), 144.8 (1C, s), 145.0 (1C, s), 145.8 (1C, s), 150.3 (1C, s), 199.8 (1C, s). MS (ESI/TOF): *m*/*z* [C_46_H_41_N_4_O]^+^ (M + H) calculated 665.33, found 665.19. Found: C, 83.07; H, 6.10; N, 8.40%. C_46_H_40_N_4_O requires C, 83.10; H, 6.06; N, 8.43%.

## Supplementary Material



## Notes

Materials and methods, NMR spectra, mass spectra, X-ray crystallographic data, and chiral HPLC chromatograms (PDF)
