# Supplementary material for: The Formylation of N,N‑Dimethylcorroles
Source: ACS Omega. 2025 Oct 1;10(40):47129–40. doi: 10.1021/acsomega.5c05661 (PMC12529165; doi:10.1021/acsomega.5c05661)
Supplement: Supplementary file 1 [file ao5c05661_si_001.pdf]

Supporting Information

The formylation of N,N-dimethylcorroles

Sara Nardis,[a,] Alessia Fata,[a] Francesco Pizzoli,[a] Greta Imbesi,[a] Greta Petrella,[a] Daniel O. Cicero,[a] Frank R. Fronczek,[b] Kevin M. Smith,[b] and Roberto Paolesse[a]*

a) Department of Chemical Sciences and Technologies, University of Rome Tor Vergata, via della Ricerca Scientifica, 00133 Rome, Italy

b) Department of Chemistry, Louisiana State University, Baton Rouge, LA, 70803, USA.

Table of Contents

Materials and Methods 2
Characterization analysis 4
UV-Vis Spectra 4
2D NMR Spectra 6
Mass Spectra 7
X ray Crystallographic Data 11
Chiral HPLC separation 12

Materials and Methods

Reagents and solvents (Aldrich) were of the highest grade available and were used without further purification. Thin-layer chromatography (TLC) was performed on Sigma-Aldrich silica gel plates. Chromatographic purification of the reaction products was accomplished by using silica gel 60 (70–230 mesh, Sigma-Aldrich, St. Louis, MO, USA) as a stationary phase. UV-vis spectra were measured on a Varian Cary 60 Spectrophotometer using CH_2Cl_2 as solvent. Electronic circular dichroism (ECD) spectra were recorded at room temperature with a JASCO J815 spectropolarimeter in a 0.5 mm quartz cell and CH_2Cl_2 solvent. NMR experiments were performed in deuterated $CHCl_3$ and recorded with a Bruker Avance spectrometer operating at 700 MHz, equipped with a 5 mm inverse TXI probe and z-axis gradients. Mass spectra were recorded on an Agilent 6520 Q-TOF instrument. Chiral HPLC enantiomers separation. The racemic mixture of compound 3 was eluted on HPLC Shimadzu LC Column (250mm X 10mm) Lux® 5 μm Cellulose-1. Isocratic (hexane:isopropanol 8:2 v:v mobile

phase and 1.0 mL/min flow were employed. The two enantiomers were eluted at tr1 = 22.34 min and tr2 = 23.54 min. Enantiomers 3 was collected after HPLC separation.

Characterization analysis

UV-Vis Spectra

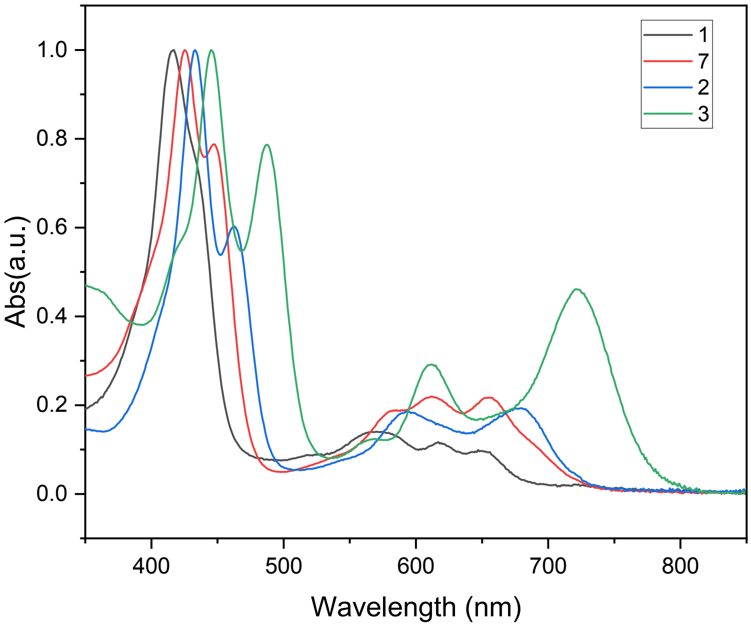

Figure S1: Comparison between the normalized UV-Vis spectra of compounds **1**, **7**, **2**, **3**, acquired in dichloromethane.

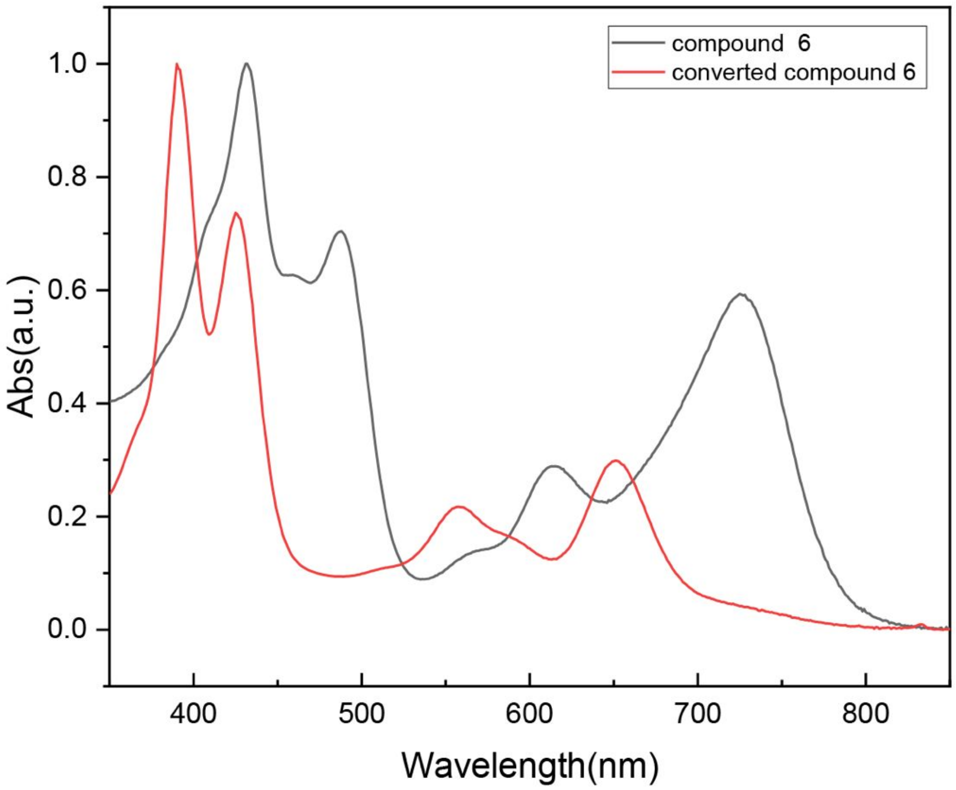

Figure S2: Comparison between the normalized UV-Vis spectra in $CHCl_3$ of compound **6** and the compound obtained after 2D NMR experiment

2D NMR Spectra

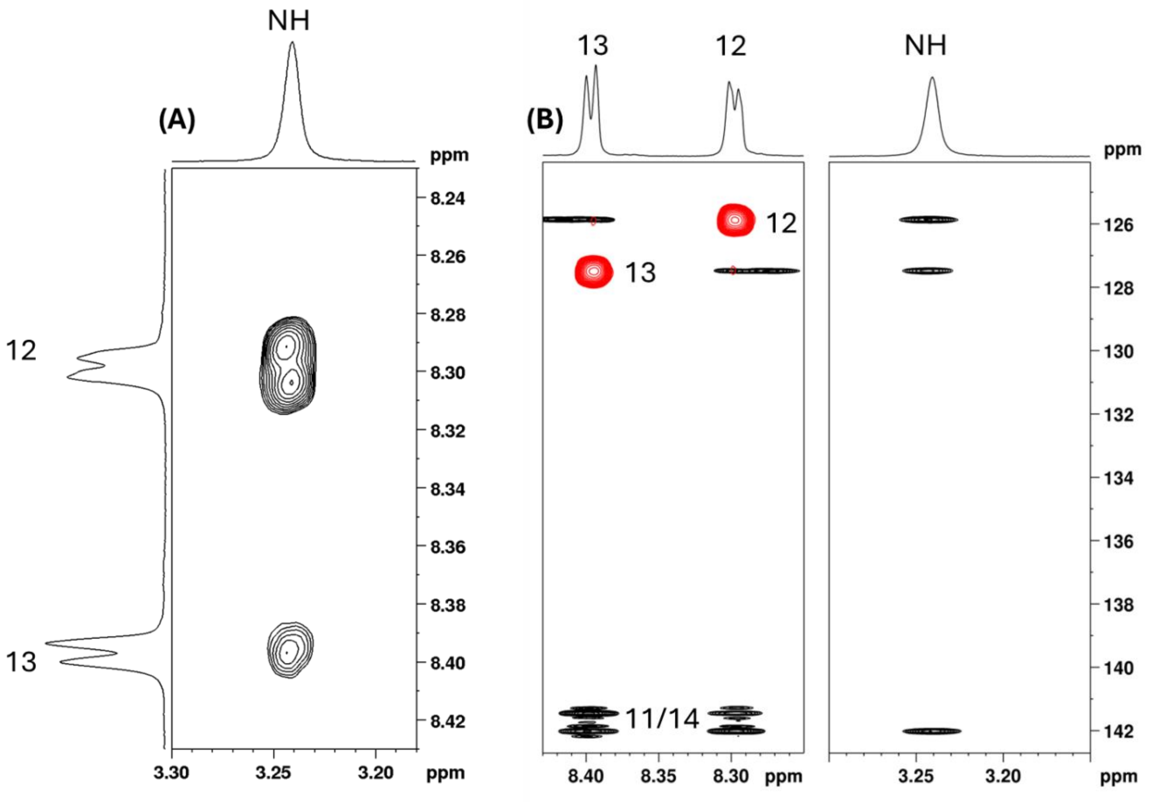

Figure S3: (A) COSY spectrum of **3** highlighting scalar correlations between the NH proton and the β-pyrrolic protons at positions 12 and 13. (B) HMBC (black) and HSQC (red) cross-peaks of the NH proton with the carbon atoms at positions 11/14 and 12/13, confirming long-range and direct NH–C connectivities. Spectra were acquired in $CDCl_3$ at 700 MHz and 283 K.

Mass Spectra

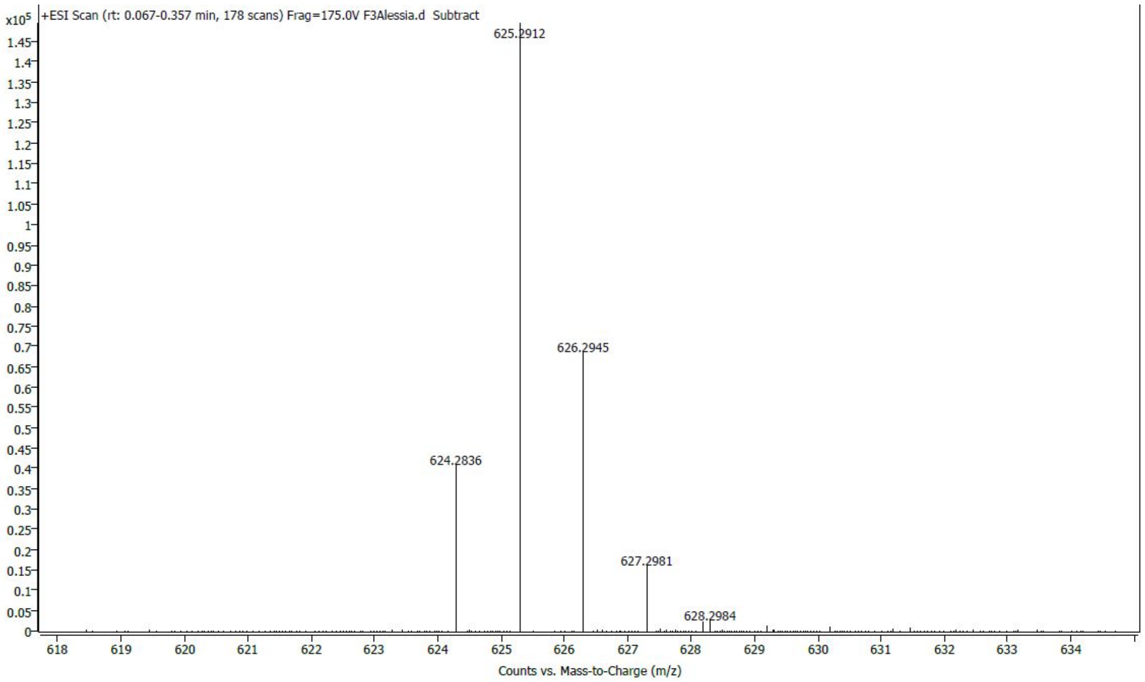

Figure S4. MS (ESI) of compound **3**.

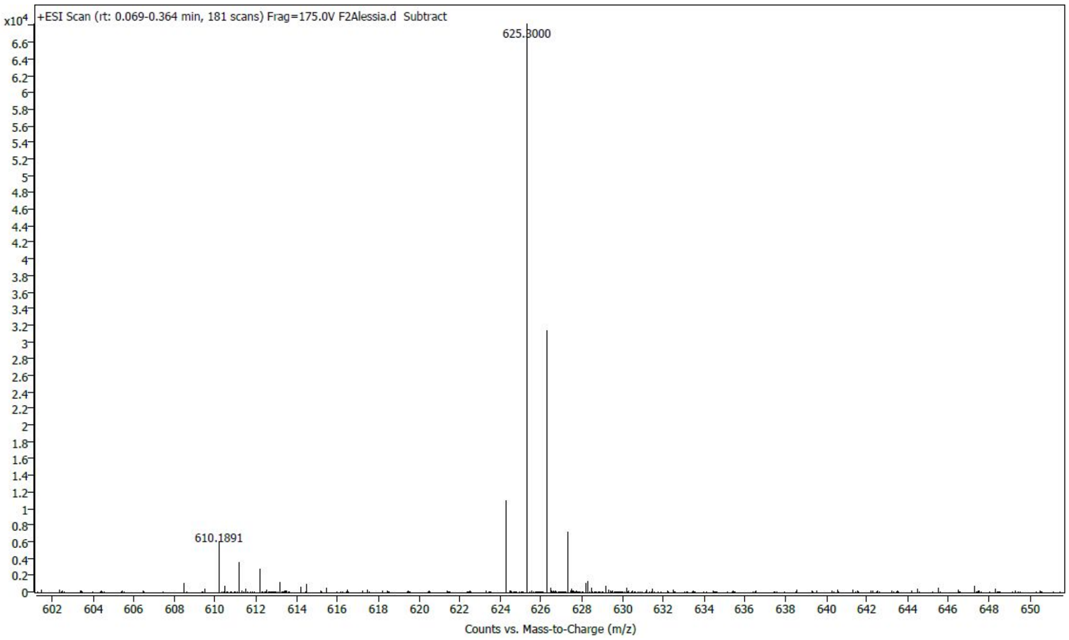

Figure S5. MS (ESI) of compound **4**.

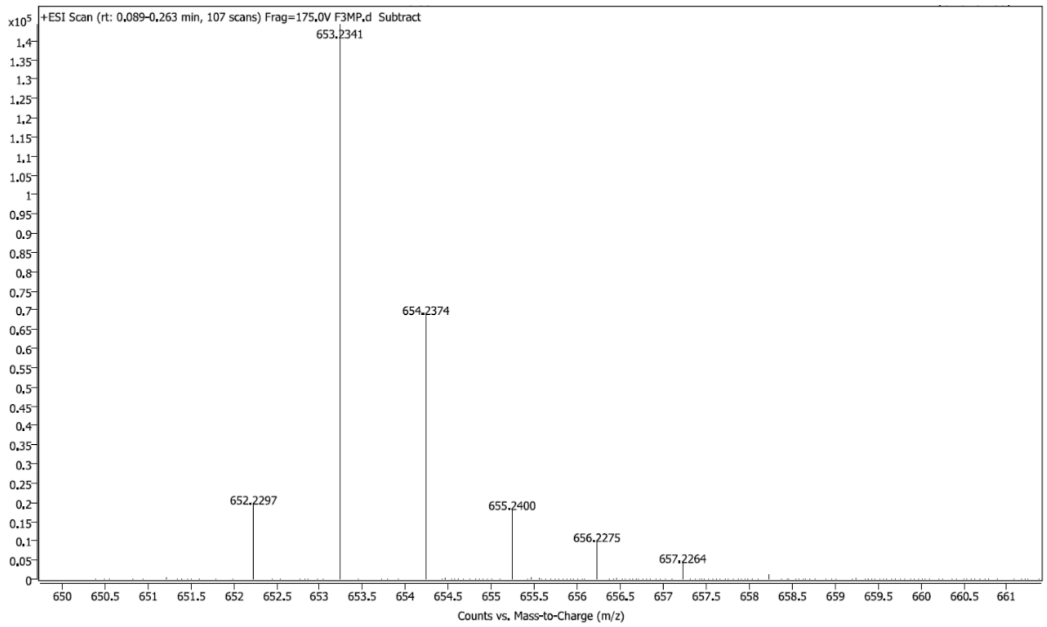

Figure S6. MS (ESI) of compound **5**.

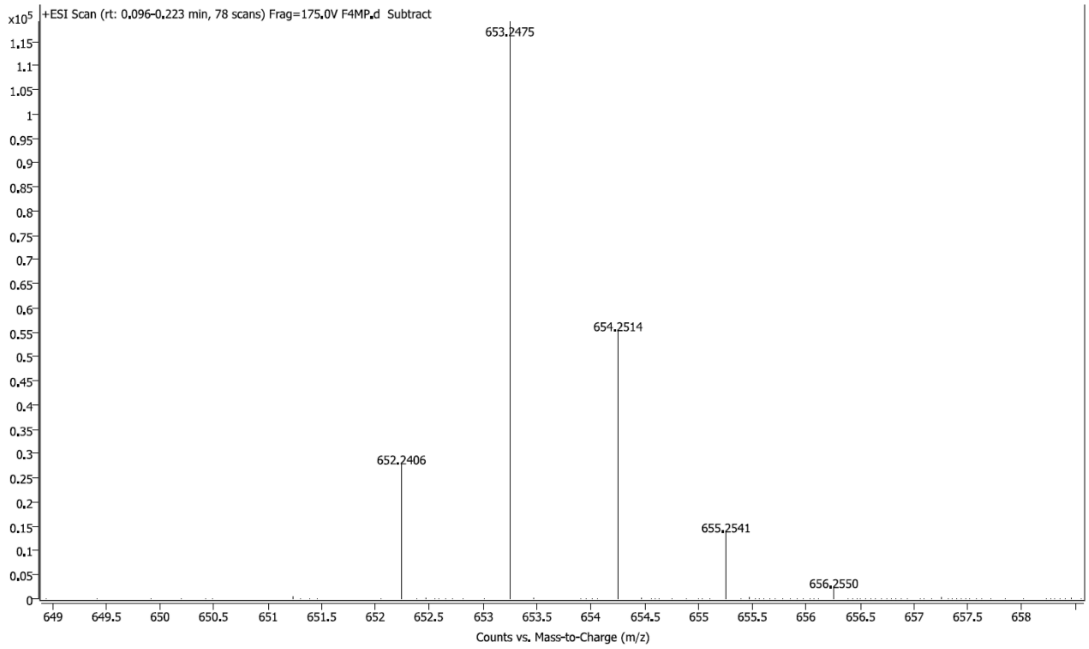

Figure S7. MS (ESI) of compound **6**.

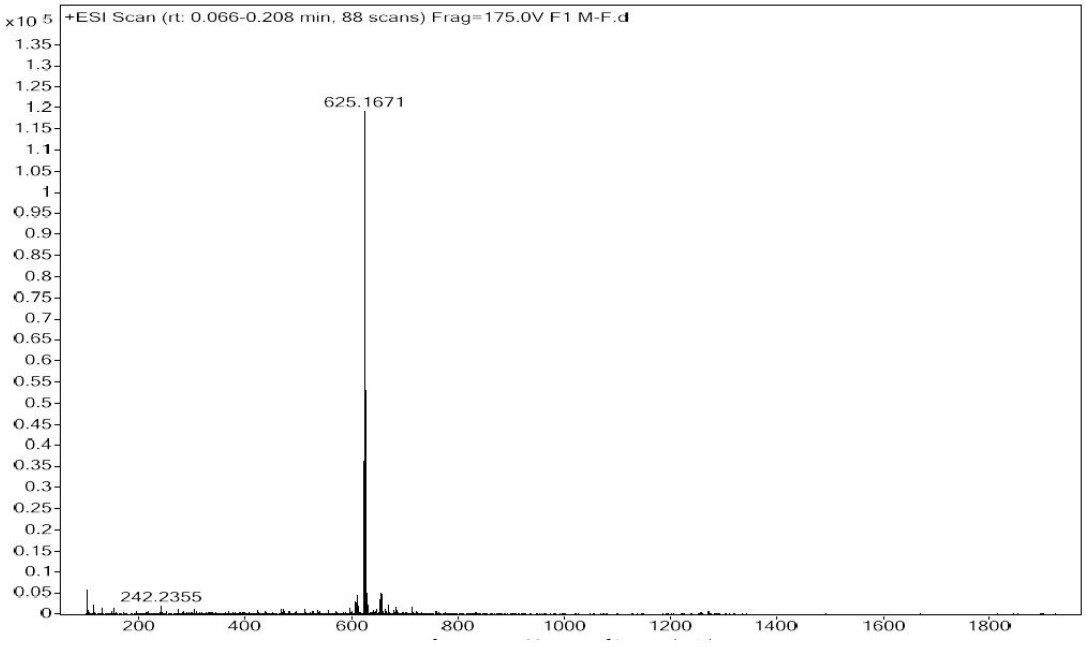

Figure S8. MS (ESI) of compound **8**.

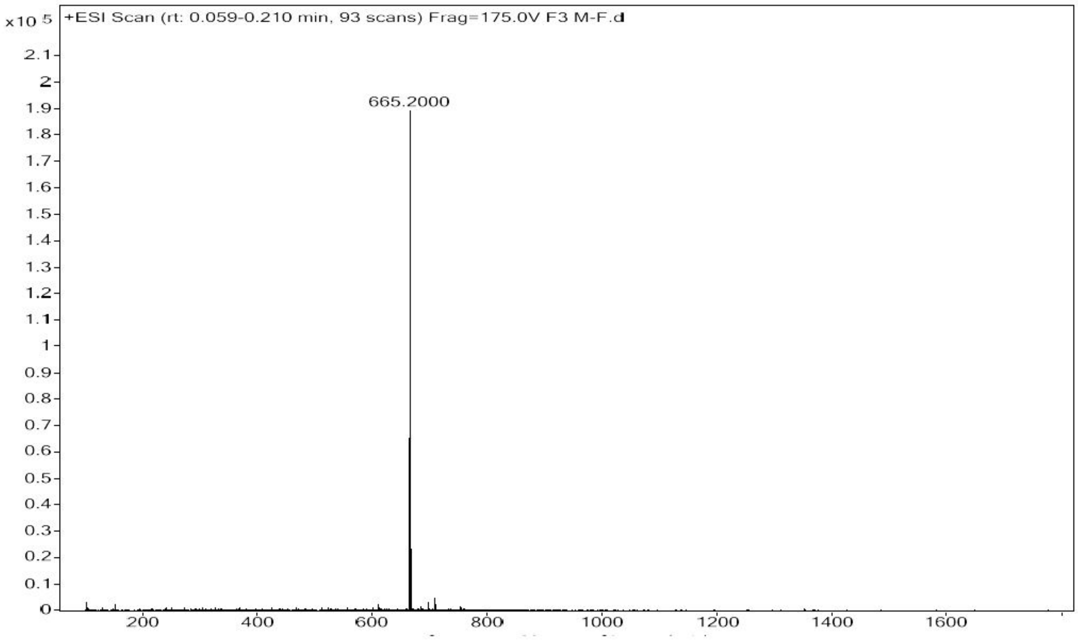

Figure S9. MS (ESI) of compound **9**.

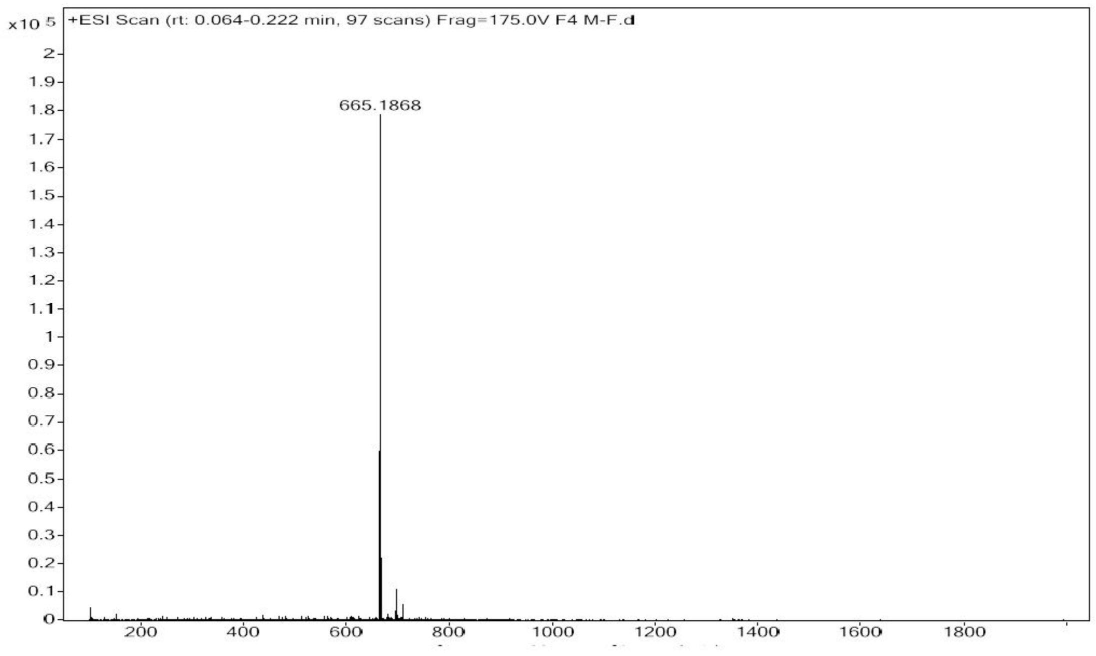

Figure S10. MS (ESI) of compound **10**.

X ray Crystallographic Data

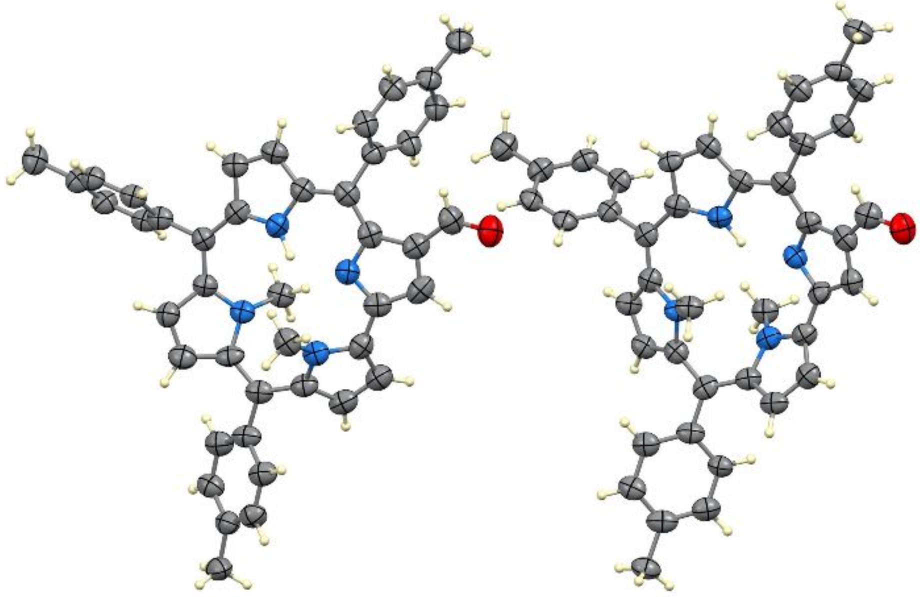

Figure S11. Crystallographic structures of **3**, evidencing both the enantiomers

Chiral HPLC separation

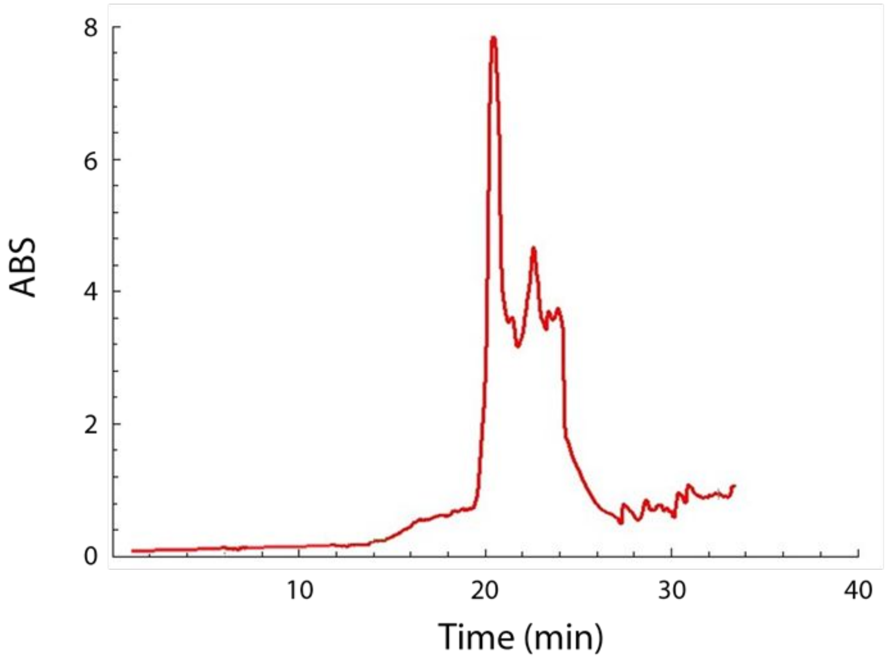

Figure S12. HPLC separation of compound **3**. Column: LC Column (250mm X 10mm) Lux® 5 mm Cellulose-1; mobile phase: Isocratic (hexane:isopropanol 8:2 v:v); flow rate: 1.0 mL min^{-1}. T = 25 ˚C. The two enantiomers were eluted at tr1 = 20.34 min and tr2 = 23.54 min.
